# JAK2/STAT3 Signaling Pathway Modulates Acute Methylmercury Toxicity in the Mouse Astrocyte C8-D1A Cell Line

**DOI:** 10.1007/s11064-025-04507-7

**Published:** 2025-08-13

**Authors:** Aafia Ahmed, Maximus Wong, Abel Santamaria, João Batista Rocha, Aaron B Bowman, Michael Aschner, Beatriz Ferrer

**Affiliations:** 1 Department of Molecular Pharmacology, Albert Einstein College of Medicine, Jack and Pearl Resnick Campus, 1300 Morris Park Avenue, Forchheimer Building, Bronx, NY 10461 USA; 2 Laboratorio de Nanotecnología y Nanomedicina, Departamento de Atención a la Salud, Universidad Autónoma Metropolitana-Xochimilco, Mexico City, 04960 Mexico; 3 Facultad de Ciencias, Universidad Nacional Autónoma de México, Mexico City, 04510 Mexico; 4 Department of Biochemical and Molecular Biology, Federal University of Santa Maria, Santa Maria, Brazil; 5 School of Health Sciences, Purdue University, West Lafayette, IN 47907 USA

**Keywords:** Methylmercury, Astrocytes, Oxidative stress, Neurotoxicity, Inflammation

## Abstract

Methylmercury (MeHg), an environmental pollutant, reaches the human body predominantly through contaminated fish consumption, potentially leading to severe neurological disorders. Upon ingestion MeHg reaches the brain and selectively accumulates in astrocytes. The activation of nuclear factor erythroid 2-related factor 2 (Nrf2) has been identified as a key early response to MeHg-induced oxidative injury, positioning it as a potential therapeutic target. However, recent studies suggest that Nrf2 activation alone may not be sufficient to mitigate MeHg toxicity, indicating the existence of other protective mechanisms. The signal transducer and activator of transcription 3 (STAT3) signaling pathway, known for its role in cell growth and survival, has emerged as a potential player in redox homeostasis. In this study, we investigated the role of STAT3 in acute (≤ 24 h) MeHg-induced neurotoxicity. MeHg exposure induced STAT3 expression in C8-D1A astrocytic cells. Our data demonstrated that pharmacological inhibition of STAT3 using AG490 or C188-9 exacerbated MeHg-induced cell death and compromised antioxidant responses. Furthermore, to fully characterize the role of STAT3 in oxidative stress, we used two different antioxidants, N-acetylcysteine (NAC) and Trolox. Conversely, reactive oxygen species (ROS)-scavenging antioxidants partially ameliorated STAT3 activation, suggesting that MeHg-induced STAT3 activation is mediated, at least in part, by mechanisms independent of ROS. Our findings suggest that STAT3 contributes to neuroprotection against MeHg exposure in astrocytes and is, at least in part, regulated by the increase in ROS levels within these cells.

**Supplementary Information:**

The online version contains supplementary material available at 10.1007/s11064-025-04507-7.

## Introduction

Mercury is naturally found within Earth’s crust [1]. For certain species like wild piscivorous fish, mammals, and birds, toxicological impacts include altered growth, reproduction, neurodevelopment, learning capacity, and behavioral abnormalities that raise mortality [2, 3]. Inorganic mercury is deposited into water bodies by attaching to airborne particles in various forms of precipitation. Microscopic organisms in water bodies combine mercury with carbon to form organic mercury (Hg). The most common type of organic mercury, methylmercury (MeHg), is found in the environment and is highly toxic [4].

MeHg can directly interact with brain and fetal cells by crossing the blood-brain and placental barriers [5]. In the gastrointestinal tract, MeHg forms a complex with L-Cysteine (CH_3_Hg-Cys) by imitating the amino acid methionine, enabling it to simply pass through the blood-brain barrier [6]. MeHg has a high affinity to sulfhydryl (-SH) protein groups, making it difficult to eliminate the compound from cells [7]. The kidneys and the nervous systems are particularly susceptible to MeHg poisoning [8].

The key mechanisms involved in MeHg-induced toxicity include increased reactive oxygen species (ROS), biomarkers of oxidative stress, and superoxide dismutase activities. The activation of antioxidant molecules may defend the central nervous system (CNS) from these neurotoxic symptoms of MeHg [9, 10]. Previously, upregulation of the nuclear factor erythroid 2-related factor 2 (Nrf2) and the downstream genes have been implicated in MeHg toxicity [11]. However, many studies demonstrate the involvement of different redox signaling in MeHg-induced neurotoxicity, including the mitogen-activated protein kinase (MAPK) cascade and Rho-associated coiled coil-forming protein kinase (ROCK) signaling [12, 13], suggesting the involvement of multiple pathways to combat MeHg toxicity.

The seven members of the signal transducer and activator of transcription (STAT) proteins (STAT1, STAT2, STAT3, STAT4, STAT5a, STAT5b, and STAT6) serve as innovative therapeutic targets for the treatment of diseases [14, 15]. STAT3 is crucial for cell cycle, cell proliferation, cell apoptosis, and the development of tumors [16]. It is activated by phosphorylation and translocated to the nucleus to participate in the regulation of gene expression. STAT3 can be regulated by upstream signaling molecules, such as Janus kinase (JAK). It then translocates to the nucleus of cells and binds to target DNA to regulate the expression of downstream proteins [14]. STAT3 acts as a downstream response protein for multiple cytokines and modulates cellular proliferation and intercellular interactions [17]. Due to its critical role in a variety of biological processes, including angiogenesis, differentiation, anti-apoptosis, proliferation, and inflammatory response, this signal transducer is the subject of extensive research.

Notably, the STAT3 signaling pathway has been poorly studied in the context of MeHg toxicity. Low concentrations of MeHg enhance ciliary neurotrophic factor (CNTF)-evoked STAT3 phosphorylation in human neuroblastoma SH-SY5Y and mouse cortical neural progenitor cells (NPCs) [18]. Additionally, persistent MeHg exposure via drinking water triggers STAT3 phosphorylation in the hypothalamus of mice [19], while prolonged exposure to MeHg-albumin conjugates increases STAT3 phosphorylation in microglial N9 cells [20]. Noteworthy, the hypothalamic neuronal cell line GT1-7 provided the first evidence of a mechanistic correlation between MeHg-induced oxidative stress and STAT3 signaling [21]. Interestingly, MeHg has also been associated with amyotrophic lateral sclerosis (ALS)-like disease [22, 23], which is characterized by elevated STAT3 protein levels [24]. Despite these findings, the precise role of STAT3 activation in MeHg toxicity, specifically within astrocytes, remains unclear.

Astrocytes play a critical role in the nervous system, maintaining neuronal homeostasis, regulating neurotransmitter levels, supporting blood-brain barrier integrity, and modulating oxidative stress responses. STAT3 is a critical regulator of astrocyte function, controlling both reactive astrogliosis and astrogenesis [25]. While astrocytes are essential for nervous system health, they are major targets of MeHg toxicity in the central nervous system. Notably, astrocytes accumulate more MeHg than neurons. This accumulation disrupts glutamate homeostasis and induces oxidative stress, ultimately compromising neuronal function [26].

Although studies have documented MeHg-induced STAT3 phosphorylation in diverse cell types, including NPCs, hypothalamic neurons, and microglia [18, 20, 21], a definitive link between STAT3 activation and MeHg-mediated astrocytic damage is lacking. This knowledge gap is particularly concerning given the crucial role astrocytes play in maintaining CNS homeostasis and their established susceptibility to MeHg exposure. To address the specific role of STAT3 in the astrocytic response to MeHg, we employed an in vitro approach using C8-D1A astrocytic cells. To dissect the STAT3 pathway’s contribution, we utilized pharmacological inhibitors: AG490, a selective inhibitor of the JAK2 tyrosine kinase [27], and C188-9, a small molecule inhibitor of STAT3 that binds to the phosphotyrosyl peptide binding site in the STAT3 Src homology 2 (SH2) domain, preventing STAT3 activation [28]. To further elucidate the role of STAT3 in oxidative stress, we treated the cells with two different antioxidants, N-acetylcysteine (NAC) and Trolox. We hypothesized that MeHg exposure leads to STAT3 phosphorylation in astrocytes, potentially linking STAT3 activation to antioxidant defense mechanisms. Conversely, we predicted that disrupting STAT3 signaling would exacerbate MeHg toxicity by increasing ROS production and promoting cellular death.

## Materials and methods

### Cell Culture

Mouse astrocytic neuronal C8-D1A cell line was obtained from the American Type Cell Culture Collection (ATCC, CRL-2541) and grown in Dulbecco’s modified Eagles’ medium (DMEM) (Gibco™, 11995040) supplemented with 10% heat-inactivated fetal bovine serum (Gibco™, 10438034) and 1% penicillin/streptomycin (Gibco™, 15140148). Cells were maintained at 37 °C with 5% CO_2_.according to the ATCC mammalian tissue culture protocol and sterile techniques. All experiments were performed using C8-D1A astrocytic cells between passages 10 and 30 to ensure phenotypic consistency. Independent experiments using different cell batches were conducted to ensure biological reproducibility.

### Methylmercury Exposure

Cells were subcultured and treated with methylmercury (II) chloride (MeHg) (Sigma-Aldrich, 442534) at 0 or 0.5, 0.1, 1, 5, 10, and 20 µM for 1, 3, 6, and 24 h unless otherwise specified. These concentrations were chosen based on our group’s previous studies [29, 30] and on literature reports. Published studies using astrocytes demonstrated that 10 µM MeHg exposure for 24 h caused an accumulation of 124ng Hg/mg protein [31]. This accumulation is compatible with the reported Hg concentration in the brain from human exposures reported to cause toxic effects [32, 33].

For the experiments with the co-exposure of MeHg and STAT3 inhibitors or antioxidants, cells were treated with 0 or 10 µM for 1, 3, 6, and 24 h unless otherwise specified.

### STAT3 Inhibitor Treatments

Cells were pre-treated for 1 h with JAK2 inhibitor AG490 (Tyrphostin B42 or 2-cyano-3-(3,4-dihydroxyphenyl)-N-(phenylmethyl)-2-propenamideor), Santa Cruz Biotechnology, sc-202046) at 0, 10, 50, or 100 µM, followed by co-treatment with 0 or 10 µM MeHg at specified times.

To evaluate if the effect was specific of STAT3 or other STATs regulated by JAK2, we also inhibited STAT3 using STAT3 Inhibitor XIII, C188-9 (CalbioChem, 573128). Cells were pre-treated for 1 h with C188-9 at 0, 3, or 30 µM, followed by co-treatment with 0 or 10 µM MeHg at specified times.

### Antioxidant Treatments

Cells were treated with the antioxidants N-acetylcysteine (NAC, Sigma-Aldrich, A8199) and Trolox (Santa Cruz Biotechnology, sc-200810) to determine whether STAT3 activation is altered by oxidative stress conditions. C8-D1A cells were pre-treated for 1 h with 0, 25, or 100 µM Trolox, a vitamin E derivative, or 1 or 5 mM of NAC, a precursor to cysteine. Cells were then co-treated with 0 or 10 µM MeHg.

### Lactate Dehydrogenase Cytotoxicity Assay

C8-D1A cells were plated in a 96-well plate. Lactate dehydrogenase (LDH) released from the cells was used to measure MeHg cytotoxicity. The released LDH oxidizes lactate to generate NADH, which reacts with iodonitrotetrazolium (INT) to form the red-colored formazan salt (Smith, Wunder et al. 2011). 24 h after seeding, cells were pre-treated for 1 h with assigned concentrations of STAT3 inhibitors or antioxidants, followed by co-exposure with MeHg. LDH release was measured in the medium using the CyQUANT LDH Cytotoxicity Assay Kit (Invitrogen, C20301), following manufacturer’s instructions. Briefly, 50 µL of the medium from each well was transferred to a new plate and 50 µL of LDH reaction mixture was added to the new plate. The plate was incubated for 30 min under a fume hood protected from light, followed by the addition of 50 µL of stop solution. Absorbance was measured at 490 nm and 680 nm using a microplate reader.

### 3-(4,5-dimethylthiazol-2-yl)-2,5-diphenyltetrazolium Bromide Cell Viability Assay

Cell viability was measured with 3-(4,5-dimethylthiazol-2-yl)-2,5-diphenyltetrazolium bromide (MTT) (Thermo Fisher Scientific, M6394). After 1 h of pre-treatment with STAT3 inhibitors or antioxidants, followed by MeHg exposure at specified times and concentrations, the medium was removed. Then, 100 µL of 0.5 µg/µL MTT in Hank’s balanced salt solution (HBSS) (Gibco™, 14175095) was added, and cells were incubated at 37℃ with 5% CO_2_ for one hour and 30 min. Following incubation, the supernatant was removed, and MTT was solubilized in dimethyl sulfoxide (DMSO) (Sigma Aldrich, D8418). Absorbance was measured at 540 nm using a microplate reader.

### Reactive Oxygen Species Production

Cellular reactive oxygen species (ROS) production was assessed using a general oxidative stress indicator CM-H_2_DCFDA (Thermo Fisher Scientific, C6827). Cells were seeded in a black 96-well microplate and labeled with 5 µM CM-H_2_DCFDA. Cells were washed with HBSS, followed by treatment with STAT3 inhibitor or antioxidants for 1 h and then MeHg exposure. Fluorescence was measured at 30 min, 1, 3, 6, and 24 h after MeHg addition at excitation 495 nm and emission 525 nm using a microplate reader.

Mitochondrial ROS production was visualized using MitoSOX (Thermo Fisher Scientific, M36008). Cells were seeded in a black 96-well microplate and labeled with 500 nM MitoSOX diluted in HBSS. Cells were treated with STAT3 inhibitors for 1 h, followed by exposure to 10 µM MeHg. Fluorescence was measured at 30 min, 1, 3, and 6 h after addition of MeHg using a microplate reader.

### Glutathione Assay

Total glutathione (GSH) and reduced GSH levels were measured using the SensoLyte^®^Total GSH Assay kit (AnnaSpec, AS-72153). C8-D1A cells were pre-treated for 1 h with STAT3 inhibitors, followed by co-exposure with 10 µM MeHg for 24 h at 37 °C with 5% CO2. Cells were collected and washed in phosphate buffer solution (PBS) (Gibco™, 10,010–023). Then, cells were lysed using freeze-thaw cycles, and protein concentration was measured using the Pierce BCA Protein Assay Kit (Thermo Fisher Scientific, 23,225). GSH was measured following the manufacturer’s protocol. GSH levels were measured at 405 nm using SpectraMax iD3 microplate reader (Molecular Devices, USA). Values are expressed in mg/mL protein.

### Interleukin 6 Quantification in Culture Medium

Cells were pre-treated 1 h with STAT3 inhibitors, followed by 10 µM MeHg co-exposure for 16 h. Interleukin 6 (IL-6) concentrations were measured by enzyme-linked immunosorbent assay (ELISA) kit (DuoSet mouse IL-6, R&D systems, DY-406), following manufacturer’s protocol.

### Western Blot

Cells were sub-cultured into 60 mm culture dishes. For whole cell protein extracts, cold radioimmunoprecipitation assay (RIPA) buffer (Sigma Aldrich, R0278) was added with phosphatase inhibitor cocktails 2 and 3 (Sigma Aldrich, P5726 and P0044, respectively) and Halt™ protease inhibitor cocktail (Thermo Fisher Scientific, 78437). The lysates were sonicated and centrifuged. The supernatant was collected, and protein concentration was measured using the Pierce BCA Protein Assay Kit (Thermo Fisher Scientific, 23225). Samples were dissolved in Laemmli sample buffer (Bio-Rad, #1610737) and boiled at 100℃ for 5 min. 20 µg of protein was resolved by 4–20% sodium dodecyl sulfate-polyacrylamide gel (SDS-PAGE, Bio-Rad #4561096). The gels were then transferred to a nitrocellulose membrane and incubated for one hour with 5% bovine serum albumin (BSA, Fisher Scientific, BP1600) in Tris buffer saline (Bio-Rad, #1706435)- 0.1% tween-20 (Promega, H5151) (TBS-T) at room temperature to block non-specific binding, followed by incubation with primary antibodies diluted in 5% BSA in TBS-T buffer overnight at 4℃. The blots were washed three times and incubated with selected horseradish peroxidase-conjugated secondary antibody. Protein bands were detected with a chemiluminescent method (Thermo Fisher Scientific 34579 or 37071). ImageJ software (NIH) was used to analyze the bands. For whole lysates, densitometry was normalized to β-actin as a housekeeping protein. Antibodies used included: mouse anti-β-actin (Sigma Aldrich, A1978), rabbit anti-phospho-STAT3 (Tyr705) (Cell Signaling Technology, #9145), mouse anti-STAT3 (Cell Signaling Technology, #9139), rabbit anti-Nrf2 (Cell Signaling Technology, #12721), mouse anti-KEAP1 (R&D systems, MAB3024), rabbit anti-SOD2 (Cell Signaling Technology, #813141), goat anti-PTP1B (R&D systems, AF3954), and mouse anti-HO1 (Cell Signaling Technology, #86806).

### Quantitative Real time PCR

Cells were cultured into 60 mm culture dishes. Total RNA was isolated using the RNeasy Mini kit (Qiagen, 74104), following the manufacturer’s instructions. RNA concentration was quantified with a Nanodrop 2000 Spectrophotometer (Thermo Fisher Scientific, ND2000) and then reverse-transcribed to cDNA using a High-Capacity cDNA Reverse Transcription kit (Applied Biosystems, 4368814) according to the manufacturer’s instructions. Gene expression was determined using TaqMan Gene Expression assay (Applied Biosystems). TaqMan probes are listed in Table 1. Gene expression was determined using the 2 − ΔΔCt method [34], and data were normalized using *Gapdh* ID: Mn99999915 as a housekeeping gene.

**Table 1:** Taqman probes for qPCR analysis

Gene Symbol	Reference Sequence	Official Full Name
*Hmox1*	Mm00516005_m1	heme oxygenase 1
*Il-18*	Mm00434226_m1	interleukin 18
*Il-6*	Mm00446190_m1	interleukin 6
*Nlrp3*	Mm00840904_m1	NLR family, pyrin domain containing 3
*Nqo1*	Mm0125356ss1_m1	NAD(P)H dehydrogenase, quinone 1
*Nfe2l2*	Mm00477784_m1	nuclear factor, erythroid derived 2, like 2
*Slc7a11*	Mm00442530_m1	solute carrier family 7 member 11
*Socs3*	Mm00545913_s1	suppressor of cytokine signaling 3
*Sod2*	Mm01313000_m1	superoxide dismutase 2
*Stat3*	Mm01219775_m1	signal transducer and activator of transcription 3
*Tgfb1*	Mm01178820_m1	transforming growth factor, beta 1
*Tnf*	Mm00443259_g1	tumor necrosis factor
*Gapdh*	Mm99999915_g1	glyceraldehyde-3-phosphatase dehydrogenase

### Statistical Analysis

Statistical analyses were conducted using IBM Statistical Package for the Social Sciences (SPSS) software version 29.0.2.0. Graphical representations were created using GraphPad Prism software version 10.4.1. No sample size calculations or outliers’ tests were performed, and no data point was excluded. Normality was assessed using the Shapiro-Wilk test and the Kolmogorov-Smirnov test.

The effects of MeHg on cytotoxicity, cell viability, ROS production, protein expression, and gene expression were assessed using one-way analysis of variance (ANOVA), followed by Bonferroni’s *post hoc* test. In cases where normality was not achieved, the data were analyzed with the non-parametric Kruskal-Wallis test, followed by Dunn’s *post hoc* test adjusted by Bonferroni correction.

The effects of MeHg on GSH levels were analyzed using Mann-Whitney U test.

The effects of STAT3 inhibitors in the presence or absence of MeHg on cytotoxicity, cell viability, oxidative stress, GSH levels, IL-6, protein expression, and gene expression were assessed using two-way ANOVA, followed by Bonferroni’s post hoc test. F values were indicated as follows: F_MeHg_ (methylmercury significant effect), F_AG_ (AG490 inhibitor significant effect), F_C188_ (C188-9 inhibitor significant effect), F_IntA_ (Methylmercury x AG490 inhibitor interaction significant effect), and F_IntC_ (Methylmercury x C188-9 inhibitor interaction significant effect).

The effects of antioxidants on MeHg-exposed cells regarding cytotoxicity, cell viability, oxidative stress, GSH levels, protein expression, and gene expression were analyzed using two-way ANOVA, followed by Bonferroni’s post hoc test. F values were indicated as follows: F_MeHg_ (methylmercury significant effect), F_NAC_ (NAC significant effect), F_Tx_ (Trolox significant effect), F_IntN_ (Methylmercury x NAC interaction significant effect), and F_IntT_ (Methylmercury x Trolox interaction significant effect).

For the two-way ANOVA analyses, when data did not meet normality assumptions, a logarithmic or square root transformation was used to achieve a closer approximation to normality. All data are reported as mean ± standard deviation (SD), and statistical significance was defined as *p* < 0.05.

## Results

### Exposure To MeHg Induces Mortality and Stress Oxidative in C8-D1A Astrocytic Cells

**Fig. 1:**
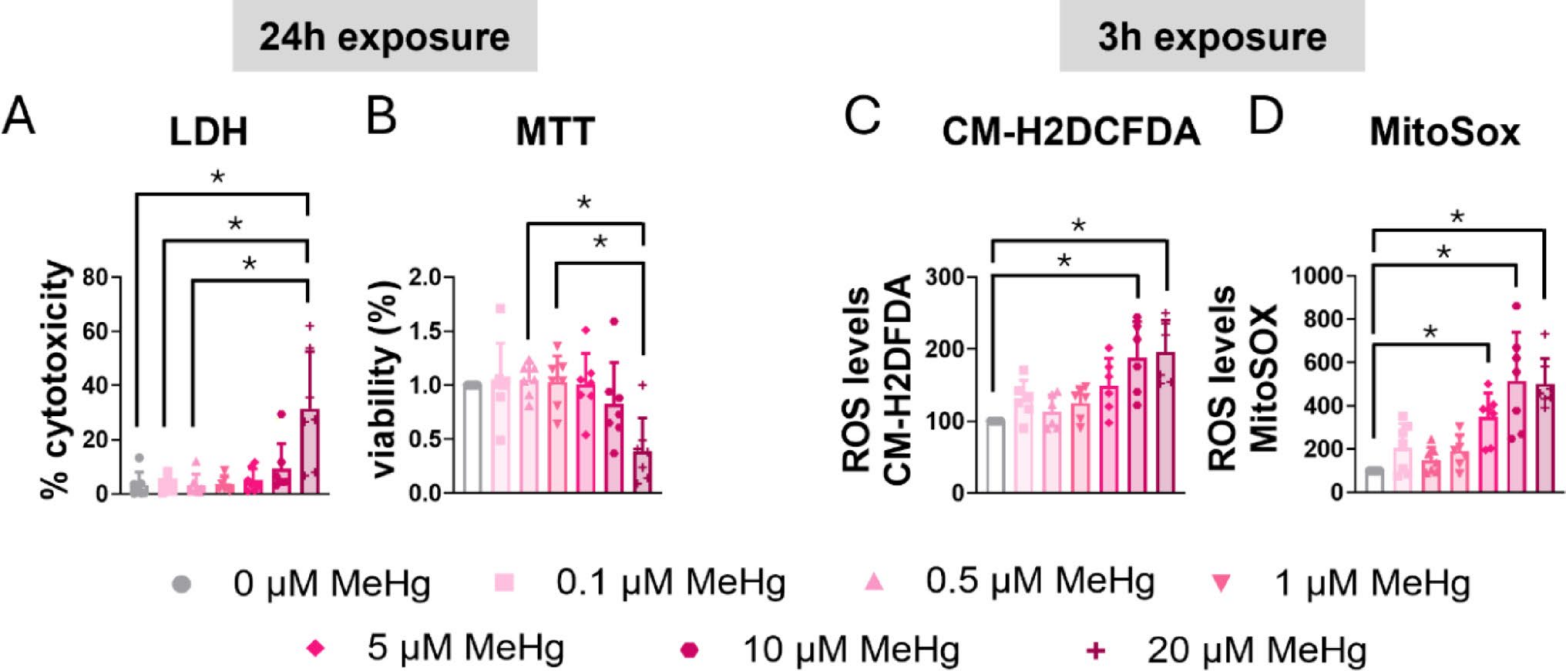
MeHg reduced cell viability and induced oxidative stress in C8-D1A astrocytic cells. Astrocytic C8-D1A cells were treated with MeHg (0, 0.1, 0.5, 1, 5, 10, or 20 µM). Cytotoxicity (**A**) and cell viability (**B**) were measured at 24 h using LDH and MTT assays, respectively. Total ROS production was measured at 3 h using the CM-H2DCFDA probe (**C**), and mitochondrial ROS production was measured using the MitoSOX probe (**D**). Data are presented as mean ± SD. Statistical significance was determined using one-way ANOVA followed by Bonferroni’s *post-hoc* analysis, or with the Kruskal-Wallis test followed by Dunn’s *post hoc* test, adjusted by Bonferroni correction when normality was not achieved. *p* < 0.05 was considered statistically significant, * denotes a significant difference

C8-D1A astrocytic cells were exposed to various concentrations of MeHg, and cytotoxicity was assessed at different time points using an LDH assay. After 24 h of exposure, the highest concentration (20 µM MeHg) significantly increased LDH release (H_(6)_ = 19.926, *p* = 0.003; *post hoc* *p* = 0.006 compared to non-exposed cells) (Fig. 1A). No significant effects were observed at shorter exposure times for any of the concentration tested (Sup. Figure 1A, B, C).

To corroborate these results, cell viability was assessed with the MTT assay. Similar to the results from the LDH assay, after 24 h of exposure, MeHg significantly reduced cell viability in a concentration dependent manner (H_(6)_ = 18.351, *p* = 0.005) (Fig. 1B). Lower concentrations and shorter exposure periods did not result in significant effects on cell viability (Sup. Figure 1D, E, F).

MeHg is known to induce oxidative stress in various cell types [35]. To assess this, we measure ROS production using CM-H2DCFDA probe, which is a general marker of intracellular ROS, in C8-D1A astrocytic cells. Twenty µM MeHg increased ROS production at 3 h of exposure (H_(6)_ = 21.927, *p* = 0.001; *post hoc* *p* = 0.006 compared to non-exposed cells) (Fig. 1C). This concentration (20 µM MeHg) also increased ROS production at 30 min (H_(6)_ = 12.897, *p* = 0.045; *post hoc* *p* = 0.013 compared to non-exposed cells) (Sup. Figure 1G) and 6 h (H_(6)_ = 27.918, *p* < 0.001; *post hoc* *p* = 0.002 compared to non-exposed cells) (Sup. Figure 1I). Interestingly, exposure to 10 µM MeHg also significantly increased ROS production at 3 h (*post hoc* *p* = 0.031 compared to non-exposed cells) (Fig. 1C). and at 6 h (*post hoc* *p* = 0.014 compared to non-exposed cells) (Sup. Figure 1I).

MeHg disrupts mitochondrial function, leading to increased ROS production, particularly superoxide, within the mitochondria [35, 36]. To investigate this, we assessed mitochondrial superoxide production in C8-D1A astrocytic cells exposed to MeHg using MitoSOX Red. MeHg significantly increased mitochondrial superoxide production at 3 h (H_(6)_ = 33.564, *p* < 0.001) (Fig. 1D). Similar results were observed at 30 min of exposure (H_(6)_ = 25.446, *p* < 0.001) (Sup. Figure 1 J), 1 h (H_(6)_ = 23.903, *p* < 0.001) (Sup. Figure 1 K), and 6 h (H_(6)_ = 22.712, *p* < 0.001) (Sup. Figure 1 L). Notably, a concentration of 5 µM MeHg was sufficient to induce a significant increase in superoxide production as early as 30 min.

To further assess the impact of MeHg on oxidative stress, we next evaluate intracellular glutathione (GSH) levels. It is well-established that MeHg induces cellular GSH depletion, which consequently reduces antioxidant capacity and exacerbates oxidative stress [37–39]. However, after 6 h of exposure to 10 µM MeHg we did not observe significant changes in total GSH levels (Sup. Figure 1 M).

### Exposure to MeHg Induces Antioxidant Enzymes Expression in C8-D1A Astrocytic Cells

**Fig. 2:**
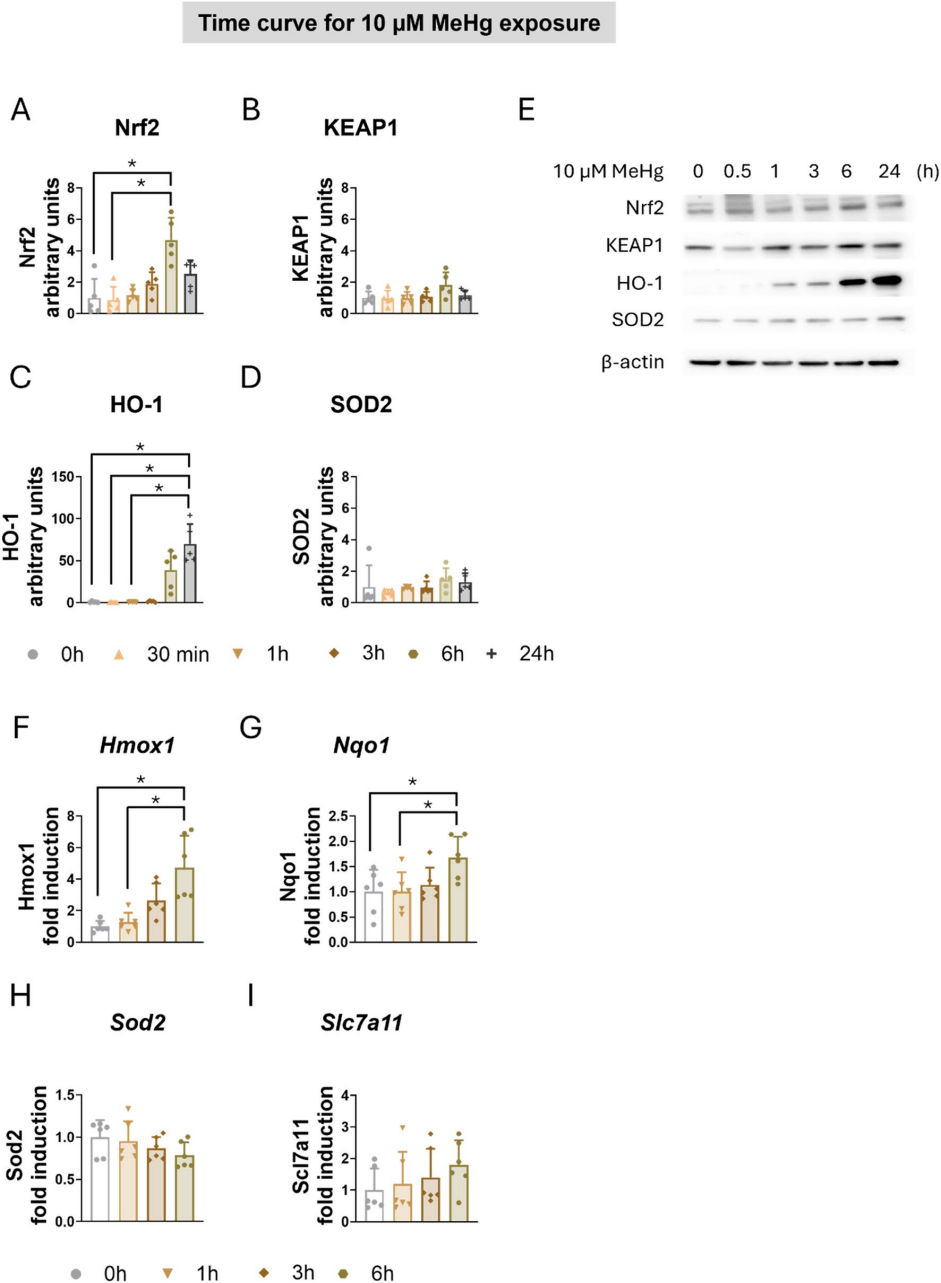
MeHg induced the expression of antioxidant enzymes in C8-D1A astrocytic cells. Astrocytic C8-D1A cells were treated with MeHg (0, 0.1, 0.5, 1, 5, 10, or 20 µM). Western blot analysis was used to measure changes in the antioxidant proteins Nrf2 (**A**), KEAP1 (**B**), HO-1 (**C**), and SOD2 (**D**) in C8-D1A cells exposed to 10 µM MeHg for 30 min, 1, 3, 6, or 24 h. (**E**) shows representative densitometry images. *Hmox1* (**F**), *Nqo1* (**G**), *Sod2* (**H**), and *Slc7a11* (**I**) gene expression were measured using qPCR in cells exposed to 10 µM MeHg for 1, 3, or 6 h. Data are presented as mean ± SD. Statistical significance was determined using one-way ANOVA followed by Bonferroni’s *post-hoc* analysis, or with the Kruskal-Wallis test followed by Dunn’s *post hoc* test, adjusted by Bonferroni correction when normality was not achieved. *p* < 0.05 was considered statistically significant, * denotes a significant difference

Under oxidative stress conditions, cellular defense mechanisms against oxidative, electrophilic, and environmental stressors are activated. MeHg activates the kelch like ECH associated protein 1 (KEAP1)/Nrf2 signaling pathway, increasing Nrf2 expression in the nucleus and upregulating the transcription of Nrf2-target genes, including Heme oxygenase 1 (HO1), NAD(P)H: quinone-oxidoreductase (NQO1), and solute carrier family 7 member 11 (SLC7a11) [29, 40–42]. In our study, MeHg significantly induced Nrf2 protein levels in both time-dependent (H_(5)_ = 18.378, *p* = 0.003) (Fig. 2A, E) and concentration-dependent manner (H_(6)_ = 24.884, *p* < 0.001) (Sup. Figure 2A, E). However, MeHg did not affect KEAP1 protein expression (Fig. 2B, E, and Sup. Figure 2B, E). To confirm the activation of this pathway, we measured HO-1 protein expression. MeHg significantly increased HO-1 protein levels in time-dependent manner (H_(5)_ = 20.680, *p* < 0.001) (Fig. 2C, E), although no significant effects were observed when C8-D1A cells were exposed to different concentrations of MeHg for 3 h (Sup. Figure 2C, E). Notably, MeHg did not affect protein levels of Superoxide dismutase 2 (SOD2), a mitochondrial antioxidant enzyme (Fig. 2D, E, and Sup. Figure 2D, E).

To further characterize the effects of MeHg on KEAP1/Nrf2 pathway, we assessed the gene expression of Nrf2-target antioxidant genes by qPCR in C8-D1A astrocytic cells exposed to 10 µM MeHg for up to 6 h. MeHg significantly induced *Hmox1* gene expression (H_(3)_ = 17.147, *p* < 0.001) (Fig. 2F) and *Nqo1* gene expression (F_(3,20)_ = 3.930, *p* = 0.023) (Fig. 2G) in a time-dependent manner, with significant effects observed at 6 h. However, no changes were detected in *Sod2* (Fig. 2H) and *Slc7a11* (Fig. 2I) mRNA expression.

### Exposure to MeHg Induces STAT3 Signaling Pathway in C8-D1A Astrocytic Cells

**Fig. 3:**
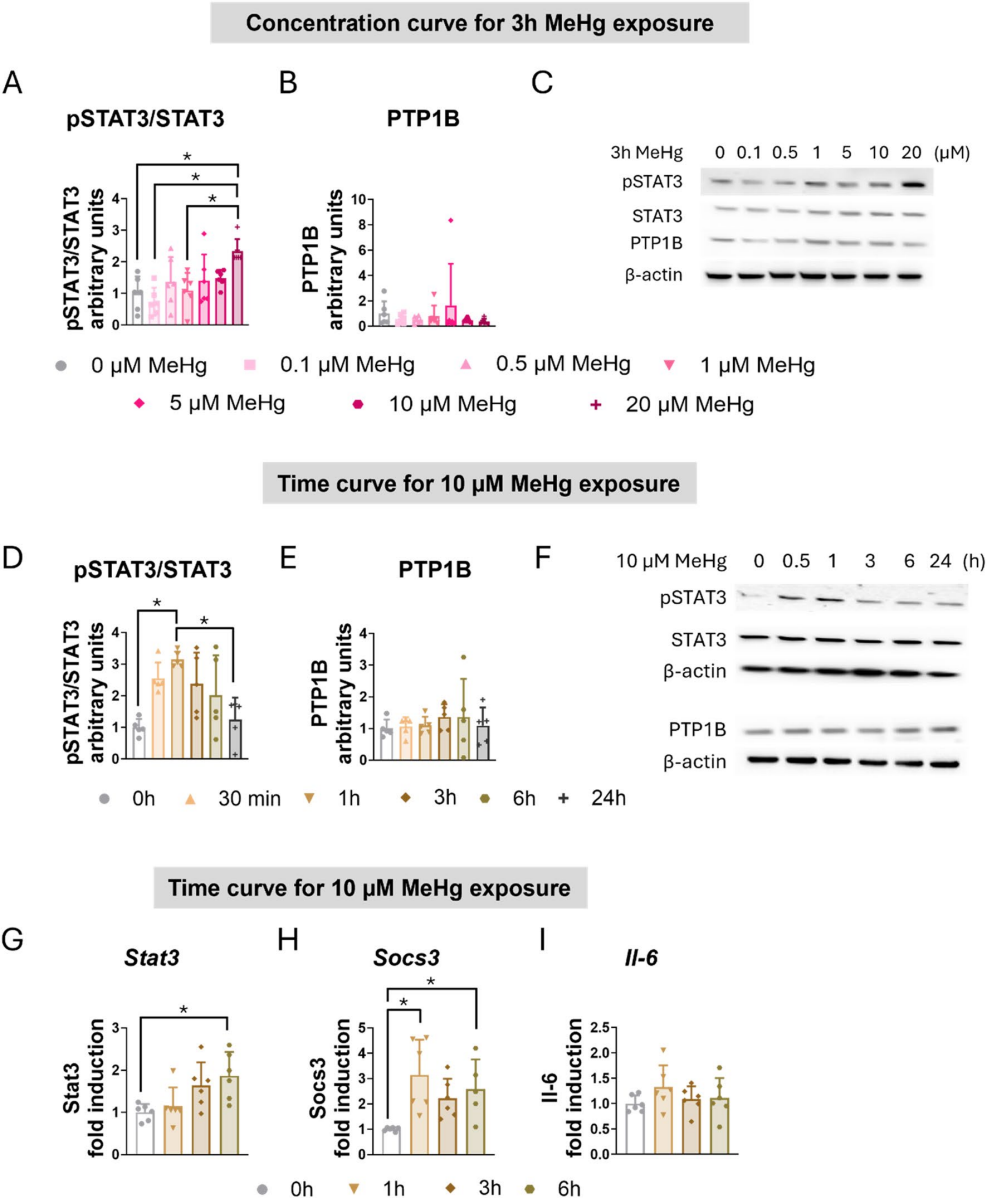
MeHg induced STAT3 activation in C8-D1A astrocytic cells. For the concentration curve, C8-D1A astrocytic cells were treated with MeHg (0, 0.1, 0.5, 1, 5, 10, or 20 µM) for 3 h. The ratio of phosphorylated to total STAT3 (**A**) was calculated, and PTP1B (**B**) protein levels were measured using western blot analysis. (**C**) shows representative densitometry images. For the time curve, C8-D1A astrocytic cells were treated with 10 µM MeHg for 30 min, 1, 3, 6, or 24 h. The ratio of phosphorylated to total STAT3 (**D**) was calculated, and PTP1B (**E**) protein levels were measured using western blot analysis. (**F**) shows representative densitometry images. *Stat3* (**G**), *Socs3* (**H**), and *Il-6* (**I**) gene expression were measured using qPCR in cells exposed to 10 µM MeHg for 1, 3, or 6 h. Data are presented as mean ± SD. Statistical significance was determined using one-way ANOVA followed by Bonferroni’s *post-hoc* analysis, or with the Kruskal-Wallis test followed by Dunn’s *post hoc* test, adjusted by Bonferroni correction when normality was not achieved. *p* < 0.05 was considered statistically significant. * denotes a significant difference

Our group has previously demonstrated that MeHg induces the STAT3 signaling pathway in hypothalamic cells [19, 21]. Next, we assessed whether MeHg increases STAT3 activation in C8-D1A astrocytic cells. Western blot analysis demonstrated that MeHg increased the ratio of phosphorylated STAT3 to total STAT3 (pSTAT3/STAT3) in a concentration-dependent (F_(6,35)_ = 4.724, *p* = 0.001) (Fig. 3A, C) and time-dependent manner (F_(5,24)_ = 5.799, *p* = 0.001) (Fig. 2D, F).

Protein Tyrosine Phosphatase 1B (PTP1B) acts as a negative regulator of the Janus Kinase 2 (JAK2)/ STAT3 signaling pathway by dephosphorylating JAK2 and STAT3 [43, 44]. However, MeHg exposure did not significantly alter PTP1B protein levels in C8-D1A astrocytic cells (Fig. 3B, C, E, F).

To further characterize MeHg-induced activation of the STAT3 signaling pathway, we evaluated the mRNA expression of various STAT3 target genes in C8-D1A astrocytic cells exposed to 10 µM MeHg for up to 6 h. MeHg significantly induced the gene expression of *Stat3* (H_(3)_ = 10.700, *p* = 0.013) (Fig. 3G) and *Socs3* (H_(3)_ = 13.555, *p* = 0.004) (Fig. 3H) in a time-dependent manner. Notably, significant *Stat3* gene induction by MeHg was observed at 6 h (*post hoc* *p* = 0.033 compared to non-exposed cells) (Fig. 2G), while significant induction of *Socs3* mRNA expression was observed at 1 h (*post hoc* *p* = 0.004 compared to non-exposed cells) and at 6 h (*post hoc* *p* = 0.036 compared to non-exposed cells) (Fig. 3H).

Given the involvement of STAT3 in inflammatory processes [45], we examined the gene expression of Interleukin 6 (*Il-6*) in C8-D1A cells exposed to 10 µM MeHg for up to 6 h. However, no significant changes in *Il-6* gene expression were detected (Fig. 3I). Similarly, exposure to MeHg did not significantly alter the expression of other inflammatory genes, including NLR family pyrin domain containing 3 (*Nlrp3)*, Interleukin 18 (*Il-18)*, Tumor Necrosis Factor (*Tnf)*, and Transforming Growth Factor Beta 1 (*Tgfb1)* (Sup. Figure 21 F, G, H, I).

### Inhibition of STAT3 Induces Death in C8-D1A Astrocytic Cells

To assess the role of STAT3 in MeHg toxicity, we used a pharmacological inhibition strategy using two different STAT3 inhibitors. This approach allowed us to discern the role of JAK2/STAT3 pathway in MeHg-induced toxicity.

**Fig. 4:**
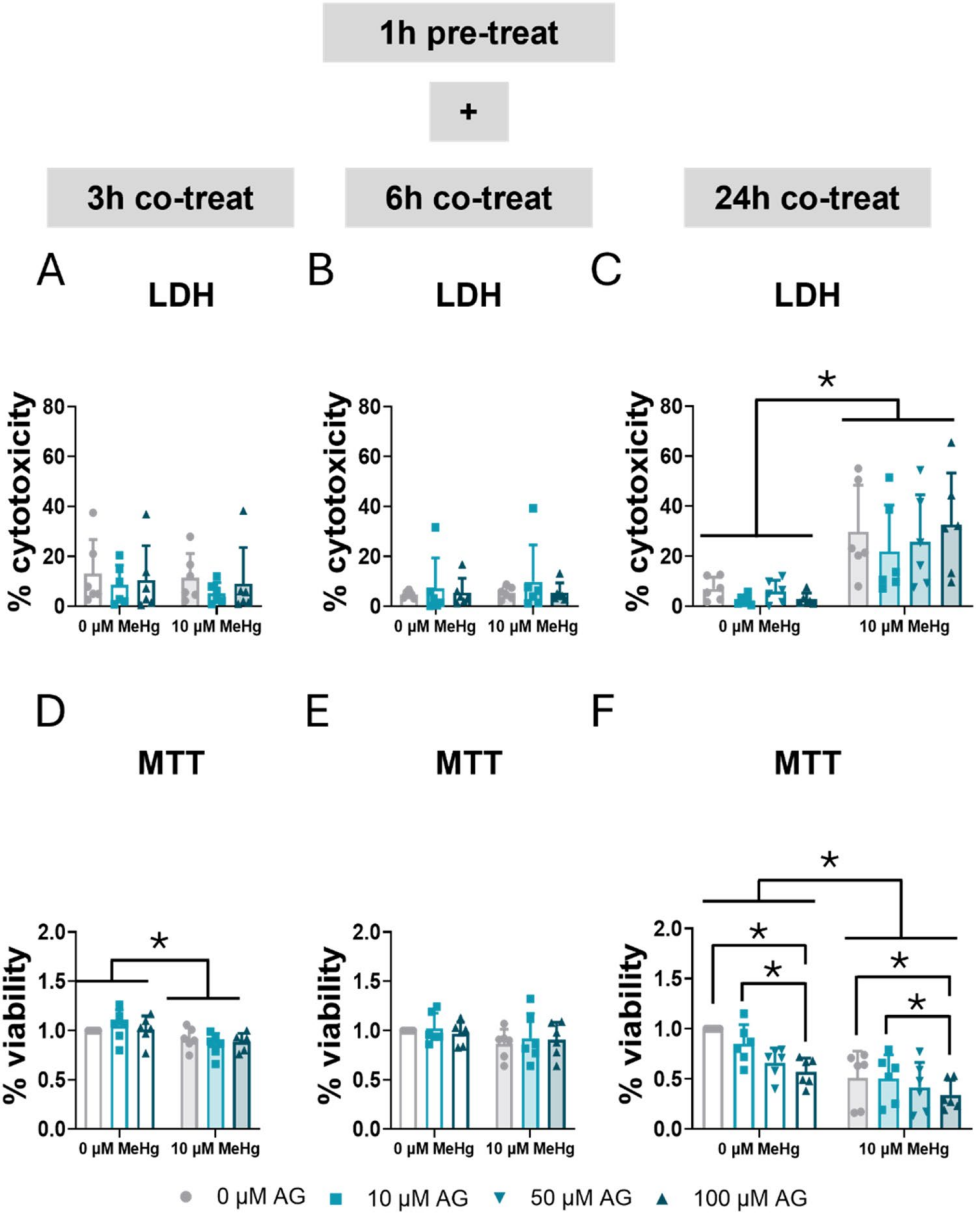
Inhibition of STAT3 phosphorylation exacerbated mortality in C8-D1A astrocytic cells. Cells were pretreated with 0, 10, 50, or 100 µM AG490, followed by co-treatment with 0 or 10 µM MeHg. Cytotoxicity (LDH release) was measured using LDH at 3 h (**A**), 6 h (**B**), and 24 h (**C**) of MeHg addition. Cell viability was assessed using MTT at 3 h (**D**), 6 h (**E**), and 24 h (**F**) of MeHg addition. Data are presented as mean ± SD. Statistical significance was determined using two-way ANOVA followed by Bonferroni’s *post-hoc* analysis. When data did not meet the assumptions of normality, a logarithmic or square root transformation was employed before conducting the two-way ANOVA. *p* < 0.05 was considered statistically significant. * denotes a significant difference

AG490 selectively blocks JAK2, inhibiting the tyrosine phosphorylation of STAT3 [27]. The impact of STAT3 inhibition on cytotoxicity and cell survival was examined. C8-D1A astrocytic cells were pre-treated for 1 h with 0, 10, 50, or 100 µM AG490, followed by co-exposure to 10 µM MeHg. Cytotoxicity was measured using an LDH assay at 3, 6, and 24 h (Fig. 4A, B, C). No significant effects were detected at 3–6 h. However, at 24 h, MeHg exposure significantly increased cytotoxicity in C8-D1A astrocytic cells across all concentrations of the inhibitor (F_MeHg(1,40)_ = 45.065, *p* < 0.001) (Fig. 4C). AG490 did not cause LDH-assay assessed cytotoxicity at any time tested (Fig. 4A, B, C).

Cell viability was assessed using an MTT assay at 3, 6, and 24 h (Fig. 4D, E, F). MeHg exposure significantly decreased cell viability at 3 h (F_MeHg(1,30)_ = 12.340, *p* = 0.001) (Fig. 4D) and 24 h (F_MeHg(1,40)_ = 35.563, *p* < 0.001) (Fig. 4F). Additionally, at 24 h, AG490 exacerbated cell death (F_AG(1,40)_ = 5.979, *p* = 0.002) (Fig. 4F).

**Fig. 5:**
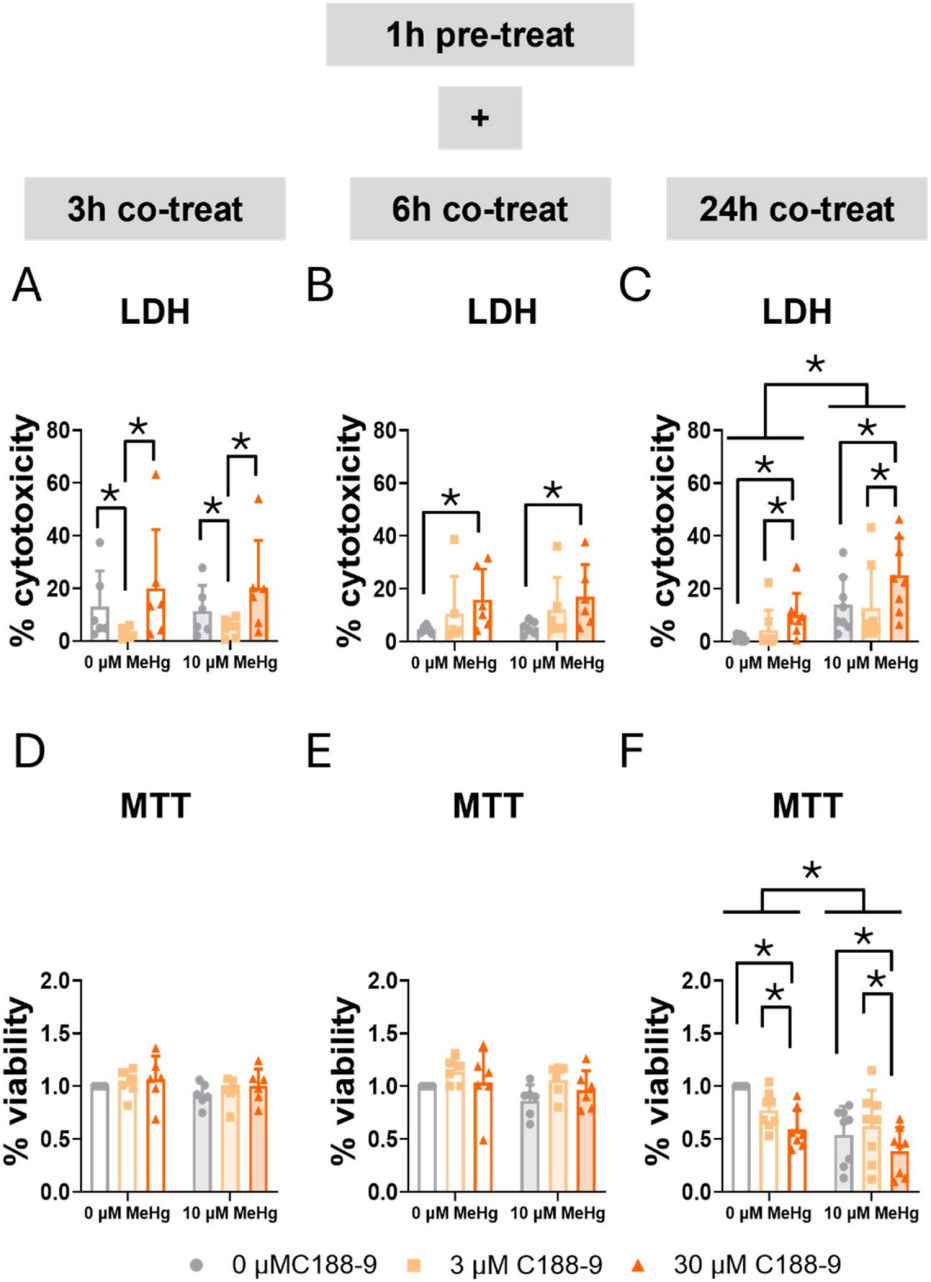
Blocking STAT3 SH2 domain increases mortality in C8-D1A astrocytic cells. Cells were pretreated with 0, 3, or 30 µM C188-9, followed by co-treatment with 0 or 10 µM MeHg. Cytotoxicity (LDH release) was measured using LDH at 3 h (**A**), 6 h (**B**), and 24 h (**C**) of MeHg addition. Cell viability was assessed using MTT at 3 h (**D**), 6 h (**E**), and 24 h (**F**) of MeHg addition. Data are presented as mean ± SD. Statistical significance was determined using two-way ANOVA followed by Bonferroni’s *post-hoc* analysis. When data did not meet the assumptions of normality, a logarithmic or square root transformation was employed before conducting the two-way ANOVA. *p* < 0.05 was considered statistically significant. * denotes a significant difference

C188-9 is a small-molecule inhibitor of STAT3. It directly inhibits STAT3 by binding to the phosphotyrosyl peptide binding site within the STAT3 SH2 domain [28, 46]. Using LDH and MTT assays, we assessed the role of STAT3 in cell survival. Consistent with our previously shown data on cell exposure to MeHg alone at different concentrations (Fig. 1 and Sup. Figure 1), no effects of MeHg were observed at early time points in the LDH assay (Fig. 5A, B) or the MTT assay (Fig. 5D, E). At 24 h, MeHg significantly increased LDH release (F_MeHg(1,42)_ = 33.035, *p* < 0.001) (Fig. 5C) and decreased tetrazolium salt (MTT) reduction (F_MeHg(1,42)_ = 17.669, *p* < 0.001) (Fig. 5F). Interestingly, the lower concentration (3 µM) of C188-9 decreased LDH release at 3 h (F_C188(2,30)_ = 7.159, *p* = 0.003; *post hoc* *p* = 0.046 compared to non-exposed cells), though this effect was not observed with the highest concentration (30 µM) (Fig. 5A). Notably, this effect disappears at 6 h, and the lethality of the high concentration becomes significant (F_C188(2,30)_ = 5.973, *p* = 0.007; *post hoc* *p* = 0.005 compared to non-exposed cells) (Fig. 5B), and it still persistent at 24 h (F_C188(2,42)_ = 8.339, *p* < 0.001; *post hoc* *p* = 0.002 compared to non-exposed cells) (Fig. 5C). In turn, the LDH assay detected an increase in mortality at early time points (6 h), the MTT assay showed a significant decrease in cell viability in the cells treated with the higher concentration (30 µM) of C188-9 at 24 h (F_C188(2,42)_ = 6.830, *p* = 0.003; *post hoc* *p* = 0.003 compared to non-exposed cells) (Fig. 5F).

LDH assay measures lactate dehydrogenase (LDH) release, an indicator of compromised membrane integrity. Conversely, the MTT assay assesses metabolic activity within mitochondria. Differences observed between LDH and MTT results suggest that STAT3 inhibition may exacerbate MeHg toxicity through different mechanisms depending on the inhibitor. Therefore, disruption of JAK2/STAT3 pathway mediates death potentially through mitochondrial dysfunction, while direct inhibition of STAT3 induces death associated with membrane integrity alterations. Moreover, C188-9 may exert a transient, potentially protective effect against cell death at an early point with lower concentration.

### Inhibition of STAT3 Phosphorylation Increases MeHg-induced ROS Production in C8-D1A Astrocytic Cells

**Fig. 6:**
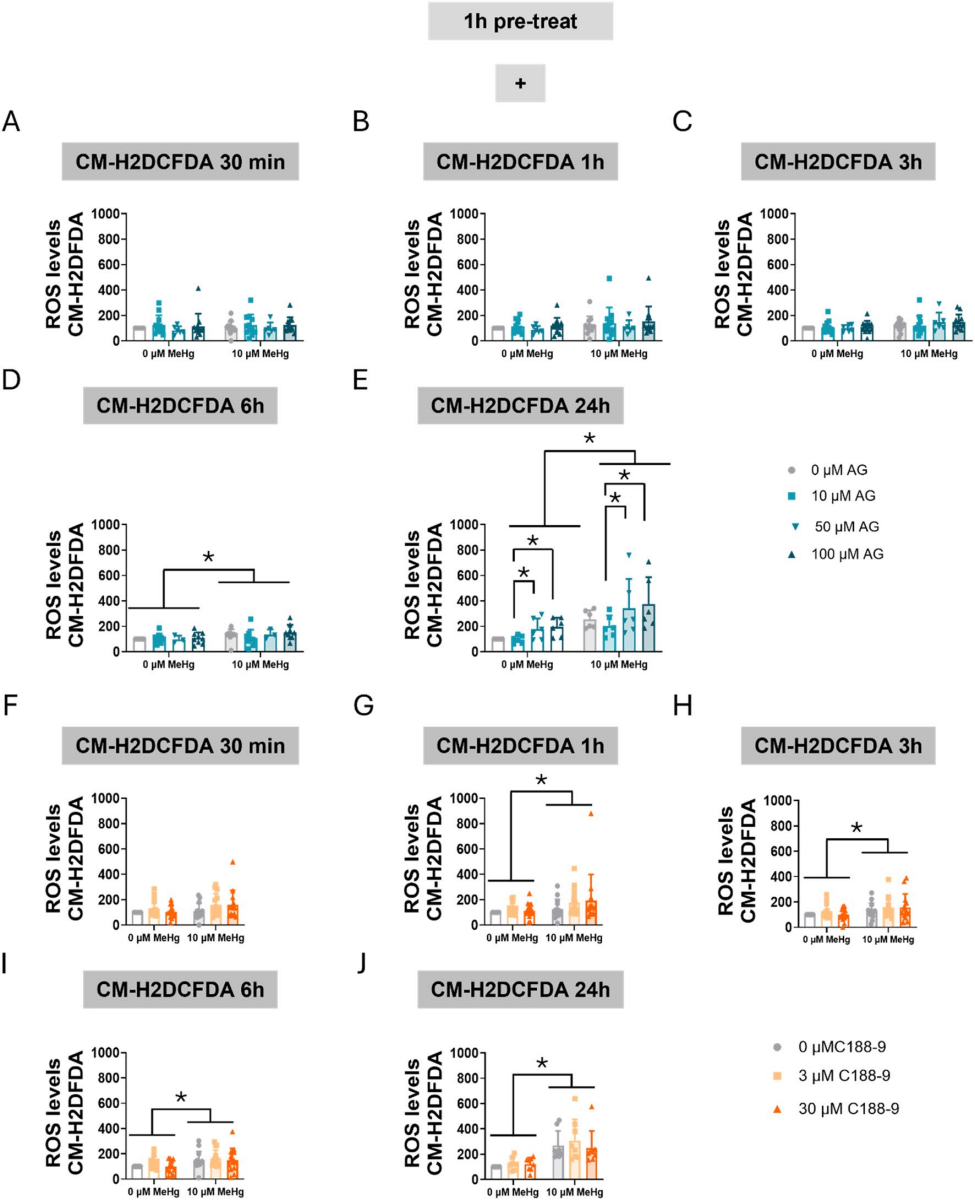
Inhibition of STAT3 phosphorylation increases oxidative stress in C8-D1A astrocytic cells. Cells were pretreated with 0, 10, 50, or 100 µM AG490 (**A**-**E**) or with 0, 3, or 30 µM C188-9 (**F**-**J**), followed by co-treatment with 0 or 10 µM MeHg. ROS production was measured using CM-H2DCFDA probe after MeHg addition at 30 min (**A**, **F**), 1 h (**B**, **G**), 3 h (**C**, **H**), 6 h (**D**, **I**), and 24 h (**E**, **J**). Data are presented as mean ± SD. Statistical significance was determined using two-way ANOVA followed by Bonferroni’s *post-hoc* analysis. When data did not meet the assumptions of normality, a logarithmic or square root transformation was employed before conducting the two-way ANOVA. *p* < 0.05 was considered statistically significant. * denotes a significant difference

The effect of STAT3 inhibition on MeHg-induced intracellular ROS production was also measured. First, we measured overall cellular ROS production using the CM-H2DCFDA probe. MeHg exposure resulted in a significant elevation of ROS levels at 6 h (F_MeHg(1,52)_ = 4.345, *p* = 0.042) (Fig. 6D), and this effect persisted at 24 h (F_MeHg(1,40)_ = 32.916, *p* < 0.001) (Fig. 6E). Interestingly, AG490 treatment induced ROS production in a concentration- dependent manner at 24 h (F_AG(3,40)_ = 5.518, *p* = 0.003) (Fig. 6E), aggravating MeHg toxicity.

When cells were pre-treated with C188-9 and then co-treated with MeHg, significant MeHg effects were detected at 1 h (F_MeHg(1,78)_ = 5.313, *p* = 0.024) (Fig. 6G), 3 h (F_MeHg(1,78)_ = 6.058, *p* = 0.016) (Fig. 6H), 6 h (F_MeHg(1,78)_ = 10.483, *p* = 0.002) (Fig. 6I), and 24 h (F_MeHg(1,42)_ = 49.003, *p* < 0.001) (Fig. 6J). Surprisingly, the C188-9 inhibitor did not affect total cellular ROS production.

**Fig. 7:**
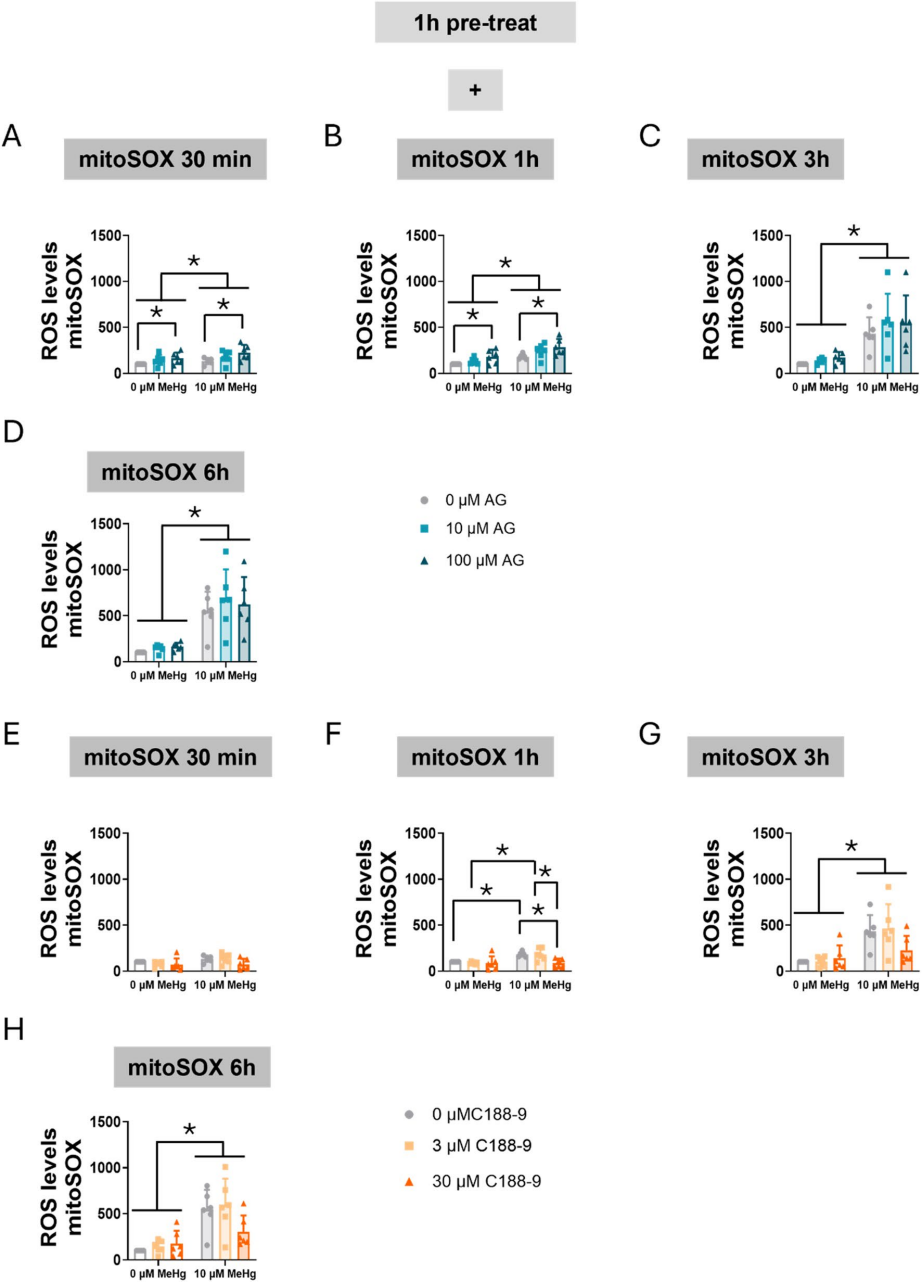
Inhibition of STAT3 phosphorylation increases mitochondrial oxidative stress in C8-D1A astrocytic cells. Cells were pretreated with 0, 10, 50, or 100 µM AG490 (**A**-**D**) or with 0, 3, or 30 µM C188-9 (**F**-**H**), followed by co-treatment with 0 or 10 µM MeHg. Mitochondrial superoxide production was assessed using mitoSOX fluorescent staining, after MeHg addition at 30 min (**A**, **E**), 1 h (**B**, **F**), 3 h (**C**, **G**), and 6 h (**D**, **H**). Data are presented as mean ± SD. Statistical significance was determined using two-way ANOVA followed by Bonferroni’s *post-hoc* analysis. When data did not meet the assumptions of normality, a logarithmic or square root transformation was employed before conducting the two-way ANOVA. *p* < 0.05 was considered statistically significant. * denotes a significant difference

Next, we assessed mitochondrial oxidative stress specifically using MitoSOX. Our data revealed that MeHg significantly increased mitochondrial superoxide levels at 30 min (F_MeHg(1,30)_ = 5.047, *p* = 0.032) (Fig. 7A), with the effect persisting at 1 h (F_MeHg(1,30)_ = 30.345, *p* < 0.001) (Fig. 7B), 3 h (F_MeHg(1,30)_ = 75.958, *p* < 0.001) (Fig. 7C), and 6 h (F_MeHg(1,30)_ = 93.996, *p* < 0.001) (Fig. 7D). Notably, AG490 increased superoxide production at 30 min (F_AG(2,30)_ = 5.460, *p* = 0.009) (Fig. 7A) and at 1 h (F_AG(2,30)_ = 7.336, *p* = 0.003) (Fig. 7B).

When cells were treated with C188-9 and MeHg the toxic compound significantly increased ROS production at 3 h (F_MeHg(1,30)_ = 31.500, *p* < 0.001) (Fig. 7G), and 6 h (F_MeHg(1,30)_ = 37.267, *p* < 0.001) (Fig. 7H). Interestingly, a significant interaction between MeHg and C188-9 was observed at 1 h (F_IntC(2,30)_ = 3.383, *p* = 0.047) (Fig. 7F). At this time point, MeHg-increased ROS levels (*post hoc* *p* = 0.005 compared to non-exposed cells) were significantly rescued at the higher (30 µM) C188-9 concentration (*post hoc* *p* = 0.004 compared with MeHg).

These findings suggest that altered STAT3 signaling contributes to modulated ROS production. AG490 potentially enhanced oxidative stress, in part through the disruption of mitochondrial homeostasis. Surprisingly, C188-9 seems to have some protective effects against MeHg-induced oxidative stress. This observation strengthens the hypothesis that STAT3 plays a key role in maintaining cellular redox balance.

### Inhibition of STAT3 Phosphorylation Decreased GSH

**Fig. 8:**
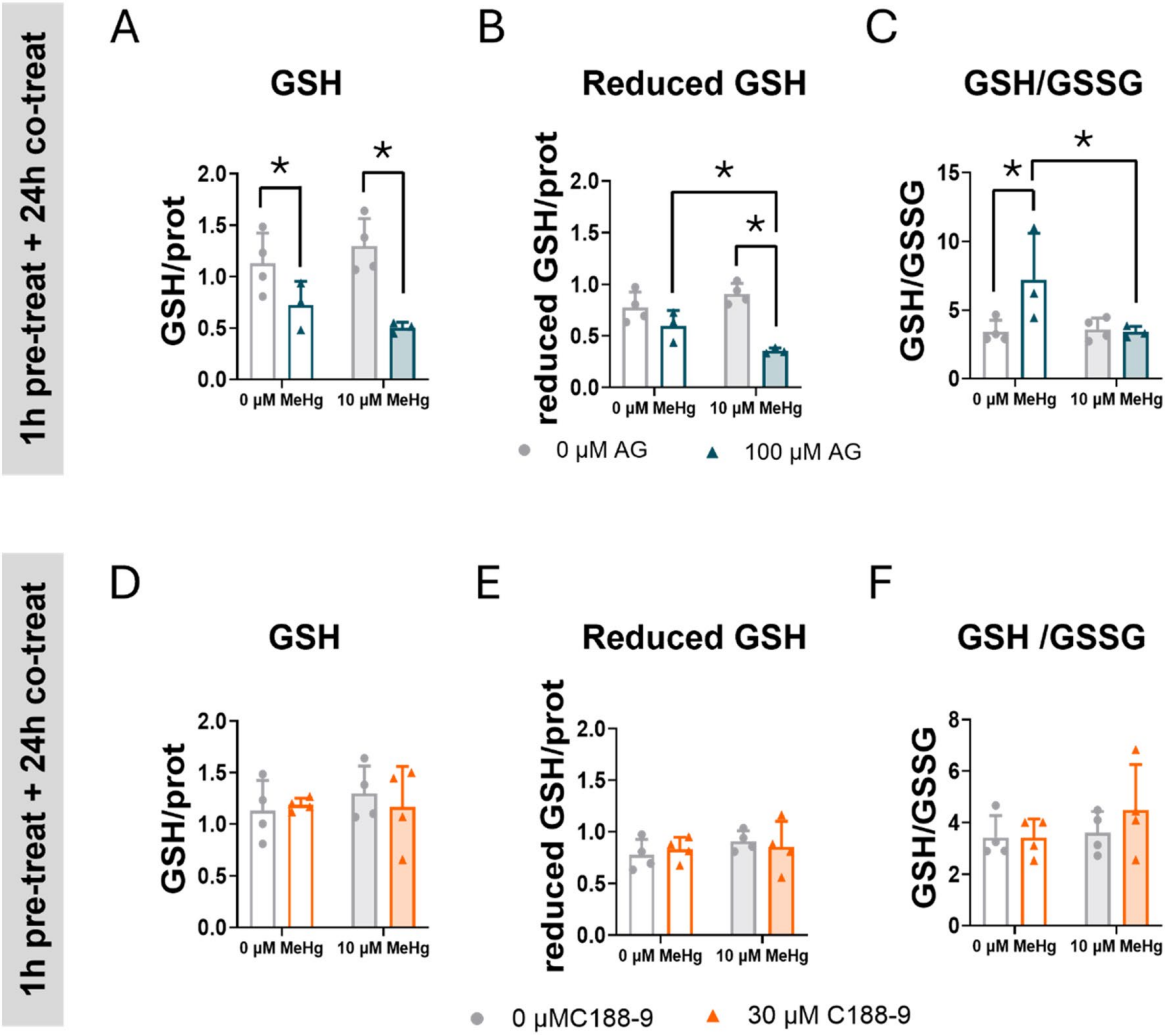
Inhibition of STAT3 phosphorylation exacerbates GSH depletion in C8-D1A astrocytic cells. Cells were pretreated with 0 or 100 µM AG490 (**A**, **B**,** C**) or with 0 or 30 µM C188-9 (**D**,** E**,** F**), followed by co-treatment with 0 or 10 µM MeHg. Total GSH (**A**, **D**) reduced GSH (**B**, **C**), and GSH/GSSG ratio were measured at 24 h after adding MeHg. Data are presented as mean ± SD. Statistical significance was determined using two-way ANOVA followed by Bonferroni’s *post-hoc* analysis. When data did not meet the assumptions of normality, a logarithmic or square root transformation was employed before conducting the two-way ANOVA. *p* < 0.05 was considered statistically significant. * denotes a significant difference

To better understand the role of STAT3 in oxidative stress, we assessed the effects of STAT3 inhibitors on GSH levels in cells exposed to MeHg. GSH is a low molecular weight thiol antioxidant compound that protects cells from oxidative stress by neutralizing ROS [38]. One of the MeHg toxic mechanisms is the depletion of GSH levels through chemical interaction between MeHg and GSH, or by impairing the GSH synthesis [38]. C8-D1A astrocytic cells were pre-treated with AG490 or C188-9, and then co-expose with 10 µM MeHg for 24 h. At the 10 µM MeHg exposure level, no significant effect of MeHg on GSH levels were detected (Fig. 8). In contrast, total GSH levels were depleted in cells treated with AG490 (F_AG(2,10)_ = 21.183, *p* < 0.001) (Fig. 8A). Interestingly, a significant interaction between MeHg and AG490 inhibitor was detected when the reduced GSH levels were measured (F_IntA(1,10)_ = 8.680, *p* = 0.015) (Fig. 8B), where MeHg exposure sensitized cells to the GSH depleting effects of AG490 (*post hoc* *p* < 0.001 compare with MeHg). When GSH levels were represented as the GSH/GSSG ratio, a significant interaction between MeHg and AG490 was observed ((F_IntA(1,10)_ = 6.165, *p* = 0.032), showing that this ratio increased in cells treated with AG490 ((*post hoc* *p* = 0.008 compared with non-treated cells). This increase was averted in cells exposed to MeHg (*post hoc p* = 0.012, compared with cells treated with AG490 and exposed to MeHg) (Fig. 8C). This change in GSH levels may explain, at least in part, the AG490-induced ROS levels (Figs. 6 and 7) observed in cells exposed to MeHg, suggesting a role for JAK2/STAT3 in redox homeostasis. Interestingly, no effects of the C188-9 inhibitor on total or reduced GSH levels were detected (Fig. 8C, D, F).

### STAT3 Inhibition Induces Aberrant Expression of Antioxidant Defense Enzymes in C8-D1A Astrocytic Cells

To fully elucidate the role of STAT3 in cellular defense against oxidative stress, cells were pre-treated 1 h with 0, 10, 50, or 100 µM AG490 or 0, 3, or 30 µM C188-9, followed by co-treatment with 0 or 10 µM MeHg at different times. Protein expression was then assessed using immunoblot. First, we determined the effects on STAT3 activation by assessing the ratio phosphorylated STAT3 to total STAT3.

**Fig. 9:**
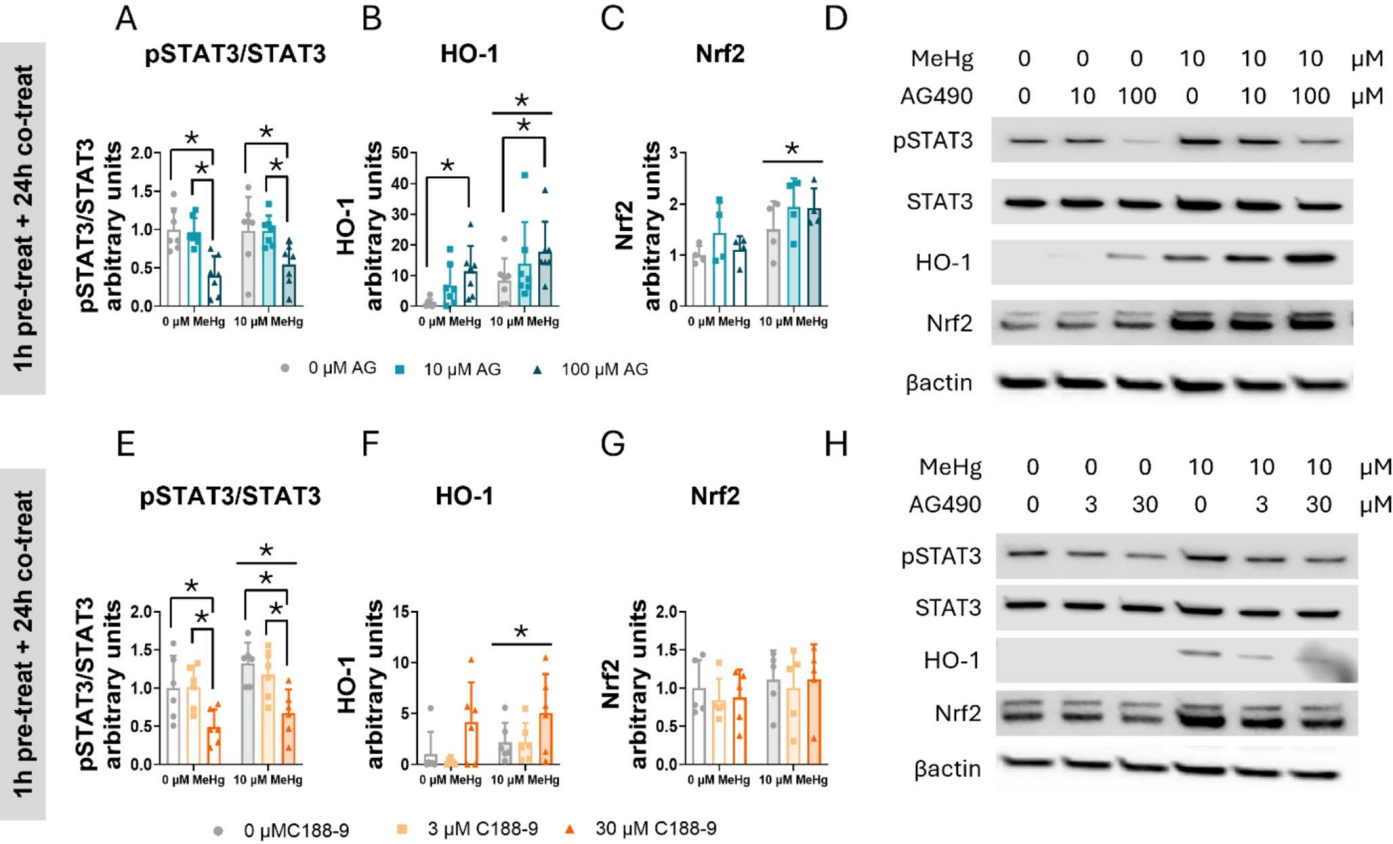
STAT3 inhibition induces aberrant antioxidant enzyme expression in C8-D1A astrocytic cells. Cells were pretreated with 0, 10, or 100 µM AG490 or with 0 or 30 µM C188-9, followed by 24 h of co-treatment with 0 or 10 µM MeHg. Protein levels of phosphorylated STAT3 to total STAT3 ratio (pSTAT3/STAT3) (**A**, **E**), HO-1 (**B**, **F**), and Nrf2 (**C**, **G**) were measured by western blot. Representative densitometry images are shown in (**D**, **H**). Data are presented as mean ± SD. Statistical significance was determined using two-way ANOVA followed by Bonferroni’s *post-hoc* analysis. When data did not meet the assumptions of normality, a logarithmic or square root transformation was employed before conducting the two-way ANOVA. *P* < 0.05 was considered statistically significant. * denotes a significant difference

In cells pre-treated with AG490 and co-exposed with MeHg, the toxic compound increased the phosphorylated STAT3 to total STAT3 ratio at 1 h (F_MeHg(1,56)_ = 190.833, *p* < 0.001) (Sup. Figure 3 A, C), 3 h (F_MeHg(1,24)_ = 49.268, *p* < 0.001) (Sup. Figure 3D, H), and 6 h (F_MeHg(1,24)_ = 16.708, *p* < 0.001) (Sup. Figure 3I, K). However, the effects of AG490 were evident at 24 h, where it decreased the phosphorylated STAT3 to total STAT3 ratio at the highest concentration tested (100 µM) (F_AG(2,36)_ = 14.789, *p* < 0.001; *post hoc* *p* < 0.001 compared to non-expose cells) (Fig. 9A, D).

In cells treated with C188-9 and co-exposed to MeHg, significant MeHg effects were detected following 24 h of exposure (F_MeHg(1,30)_ = 4.704, *p* = 0.038) (Fig. 9E, H). This effect was also evident at 3 h (F_MeHg(1,24)_ = 11.653, *p* = 0.002) (Sup. Figure 4 A, E). The highest C188-9 concentration (30 µM) significantly decreased the ratio phosphorylated STAT3 to total STAT3, indicating decreased STAT3 activation at 24 h (F_C188(2,30)_ = 12.513, *p* < 0.001; *post hoc* *p* < 0.001 compared with non-exposed cells) (Fig. 9E, H). This effect began to be evident at 6 h, although it did not reach statistical significance (F_C188(2,12)_ = 3.868, *p* = 0.051) (Sup. Figure 4 F, H).

We also examined protein tyrosine phosphatase 1B (PTP1B), a negative regulator of JAK2/STAT3 signaling pathway [47]. Neither MeHg nor AG490 or C188-9 treatment affected PTP1B protein expression (Sup. Figure 4E, H, and Sup. Figure 5B, E).

Heme oxygenase-1 (HO-1), a key antioxidant enzyme regulated by the Nrf2 pathway, is responsible for heme group degradation [48]. MeHg exposure increased HO-1 protein levels at 24 h (F_MeHg(1,36)_ = 11.095, *p* = 0.002) (Fig. 9B, D). Similar effects were observed at 3 h (F_MeHg(1,24)_ = 5.550, *p* = 0.027) (Sup. Figure 3 F, H), and at 6 h (F_MeHg(1,24)_ = 11.291, *p* = 0.003) (Sup. Figure 3 J, K). Contrary to what was observed with the phosphorylated STAT3 to total STAT3 ratio, AG490 increased HO-1 protein levels as early as 1 h at the 50 µM concentration (F_AG(2,56)_ = 2.917, *p* = 0.042; *post hoc* *p* = 0.036 compared to non-exposed cells) (Sup. Figure 3B, C), suggesting a direct link between activation of the JAK2 pathway and the Nrf2 signaling pathway. Similar to the 1 h treatment, AG490 increased HO-1 protein expression in a concentration-dependent manner at 3 h (F_AG(2,24)_ = 13.013, *p* < 0.001) (Sup. Figure 3 F, H), and at 6 h (F_AG(2,24)_ = 42.454, *p* < 0.001) (Sup. Figure 3 J, K). At 24 h, only the highest concentration (100 µM) significantly increased HO-1 protein levels (F_AG(2,36)_ = 8.330, *p* = 0.001; *post hoc* *p* < 0.001 compared to non-exposed cells) (Fig. 9B, D), indicating sustained HO-1 activation by the AG490 inhibitor.

In cells pre-treated with C188-9, MeHg significantly increased HO-1 expression at 24 h (F_MeHg(1,30)_ = 12.250, *p* = 0.001) (Fig. 9F, H). This activation was also observed after 6 h of exposure (F_MeHg(1,12)_ = 22.242, *p* < 0.001) (Sup. Figure 4G, H). Interestingly, 30 µM C188-9 enhanced the HO-1 protein levels at 3 h (F_C188(2,24)_ = 12.601, *p* < 0.001; *post hoc* *p* < 0.001 compared to non-exposed cells) (Sup. Figure 4 C, E) and 6 h (F_C188(2,12)_ = 38.053, *p* < 0.001; *post hoc* *p* < 0.001 compared to non-exposed cells) (Sup. Figure 4G, H).

To gain further insight into the mechanism by which HO-1 is expressed, we quantified Nrf2 protein levels, the transcription factor responsible for HO-1 gene expression. In cells pre-treated with AG490 and co-exposed to MeHg, Nrf2 expression was significantly upregulated at 24 h post-exposure (F_MeHg(1,18)_ = 10.882, *p* = 0.004) (Fig. 9C, D). A similar effect was also observed at the earlier 3-hour time point (F_MeHg(1,24)_ = 17.026, *p* < 0.001) (Sup. Figure 3G, H). No differences are detected at 24 h of exposure (Fig. 9G, H).

However, in cells pre-treated with C188-9, 3 h after co-exposed to MeHg Nrf2 significantly increased (F_MeHg(1,24)_ = 7.462, *p* = 0.012) (Sup. Figure 4D, E). However, Notably, no effects oAG490 or C188-9 inhibitors were detected in any of the time points measured.

**Fig. 10:**
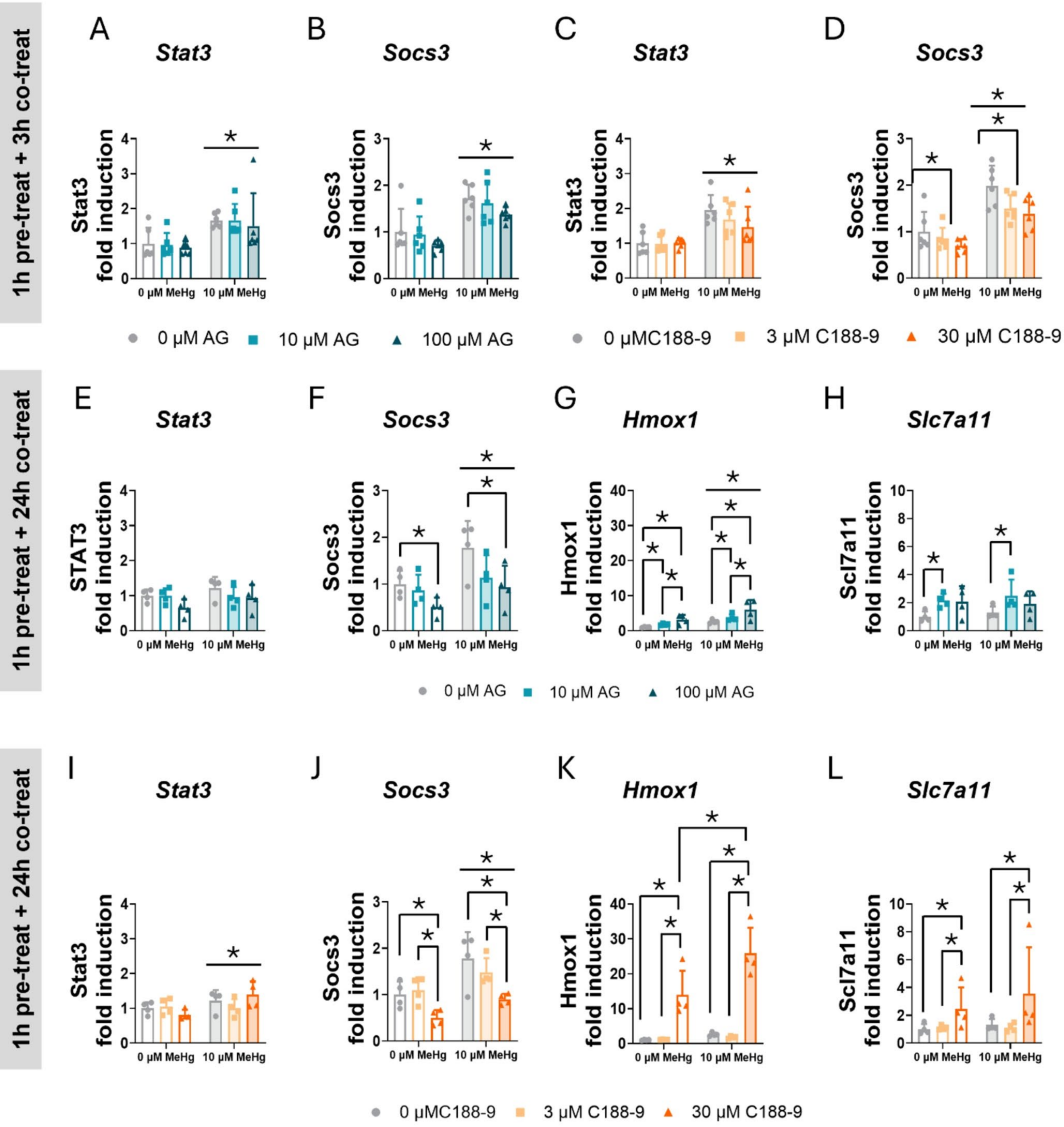
STAT3 inhibition induces aberrant antioxidant gene expression in C8-D1A astrocytic cells. Cells were pretreated with 0, 10, or 100 µM AG490 or with 0 or 30 µM C188-9, followed by 24 h of co-treatment with 0 or 10 µM MeHg. Gene expression of *Stat3* (**A**, **C**) and *Socs3* (**B**, **D**) at 3 h, and *Stat3* (**E**, **I**), *Socs3* (**F**, **J**), *Hmox1* (**G**, **K**), and *Slc7a11* (**H**, **L**) at 24 h was measured by qPCR. Data are presented as mean ± SD. Statistical significance was determined using two-way ANOVA followed by Bonferroni’s *post-hoc* analysis. When data did not meet the assumptions of normality, a logarithmic or square root transformation was employed before conducting the two-way ANOVA. *p* < 0.05 was considered statistically significant. * denotes a significant difference

To confirm that MeHg-induced STAT3 activation, we measured the expression of some STAT3-target genes using qPCR, such as *Stat3* and *Socs3*. In cells pre-treated 1 h with the AG490 inhibitor and co-exposed to MeHg, no effects of AG490 were detected on *Stat3* mRNA expression. However, MeHg significantly increased its expression at 3 h of exposure (F_MeHg(1,30)_ = 25.387, *p* < 0.001) (Fig. 10A). At 3 h, MeHg also significantly increased *Socs3* mRNA expression (F_MeHg(1,30)_ = 35.156, *p* < 0.001) (Fig. 10B). This increase was sustained at 24 h (F_MeHg(1,18)_ = 8.605, *p* = 0.009) (Fig. 10F), suggesting that MeHg induced STAT3 transcriptional activities. AG490 decreased *Socs3* gene expression, with significant effects observed at 24 h (F_AG(2,18)_ = 5.440, *p* = 0.014) (Fig. 10F).

When STAT3 activity was inhibited with C188-9, no effects of C188-9 inhibitor were detected on *Stat3* mRNA expression. However, MeHg significantly increased *Stat3* gene expression at 3 h (F_MeHg(1,30)_ = 35.706, *p* < 0.001) (Fig. 10C) and at 24 h (F_MeHg(1,18)_ = 5.618, *p* = 0.029) (Fig. 10I). MeHg also induced *Socs3* mRNA at 3 h (F_MeHg(1,30)_ = 59.075, *p* < 0.001) (Fig. 10D) and at 24 h (F_MeHg(1,18)_ = 15.694, *p* < 0.001) (Fig. 10J). The C188-9 inhibitor decreased *Socs3* mRNA expression at 3 h (F_C188(2,30)_ = 5.257, *p* = 0.011) (Fig. 10D) and at 24 h (F_C188(2,18)_ = 10.592, *p* < 0.001) (Fig. 10J), indicating that STAT3 transcriptional activity was inhibited.

To further confirm that the inhibition of STAT3 mediates the activation of antioxidant defense, we measured the gene expression of various antioxidant genes using qPCR. As expected, at 24 h, MeHg significantly increased *Hmox1* mRNA expression (F_MeHg(1,18)_ = 32.115, *p* < 0.001) (Fig. 10G), and AG490 further activated its expression (F_AG(2,18)_ = 15.764, *p* < 0.001) (Fig. 10G). Notably, these effects were observed at 3 h, where MeHg significantly increased *Hmox1* gene expression (F_MeHg(1,30)_ = 20.727, *p* < 0.001) (Sup. Figure 5 A) and this effect was enhanced by AG490 treatment (F_AG(2,30)_ = 24.767, *p* < 0.001) (Sup. Figure 5 A). At 24 h, we also assessed *Slc7a11* gene expression. Although MeHg did not induce its expression, the lowest concentration (10 µM) of AG490 upregulated it (F_AG(2,18)_ = 4.524, *p* = 0.026; *post hoc* *p* = 0.0028 compared to non-exposed cells) (Fig. 10H). In contrast, at 3 h of co-exposure, neither MeHg nor AG490 affected *Nqo1* and *Sod2* mRNA expression (Sup. Figure 5B, C).

In cells pre-treated with C188-9 and co-exposed with MeHg, a significant interaction was observed in the expression of *Hmox1* mRNA at 24 h of co-exposure (F_IntC(2,18)_ = 4.623, *p* = 0.024) (Fig. 10K). The highest C188-9 concentration (30 µM) enhanced MeHg-induced *Hmox1* gene expression (*post hoc* *p* < 0.001 compared to MeHg, and *p* < 0.001 compared to 30 µM C188-9 alone). Similar effects were observed at 3 h, where MeHg induced *Hmox1* gene expression (F_MeHg(1,30)_ = 28.946, *p* < 0.001) (Sup. Figure 5 F), and C188-9 further upregulated it (F_C188(2,30)_ = 21.500, *p* < 0.001) (Sup. Figure 5 F). Additionally, we measured *Slc7a11* mRNA expression at 24 h. While no effect of MeHg was detected, C188-9 upregulated *Slc7a11* mRNA expression (F_C188(2,18)_ = 8.375, *p* = 0.003) (Fig. 10L). In contrast, at 3 h, MeHg did not significantly affect *Nqo1* and *Sod2* gene expression (Sup. Figure 5G, H). However, C188-9 induced *Nqo1* mRNA expression at 3 h (F_C188(2,30)_ = 21.702, *p* < 0.001) (Sup. Figure 5G). Finally, *Nfe2l2* gene expression, which encodes NRF2, was not affected by MeHg, AG490, or C188-9 (Sup. Figure 5D, I).

Altogether, these data suggest that STAT3 inhibitors may mediate activation of antioxidant defense.

### STAT3 Inhibition Did not Prevent MeHg-induced Interleukin 6 Release in C8-D1A Astrocytic Cells

Due to the crucial role of STAT3 in neuroinflammation [49, 50] we measured the expression of some inflammatory cytokines. Although it has been reported that MeHg induces *Tnf* gene expression [51], we did not detect any significant effect. Neither AG490 nor C188-9 affected *Tnf* mRNA expression (Sup. Figure 5E, J).

**Fig. 11:**
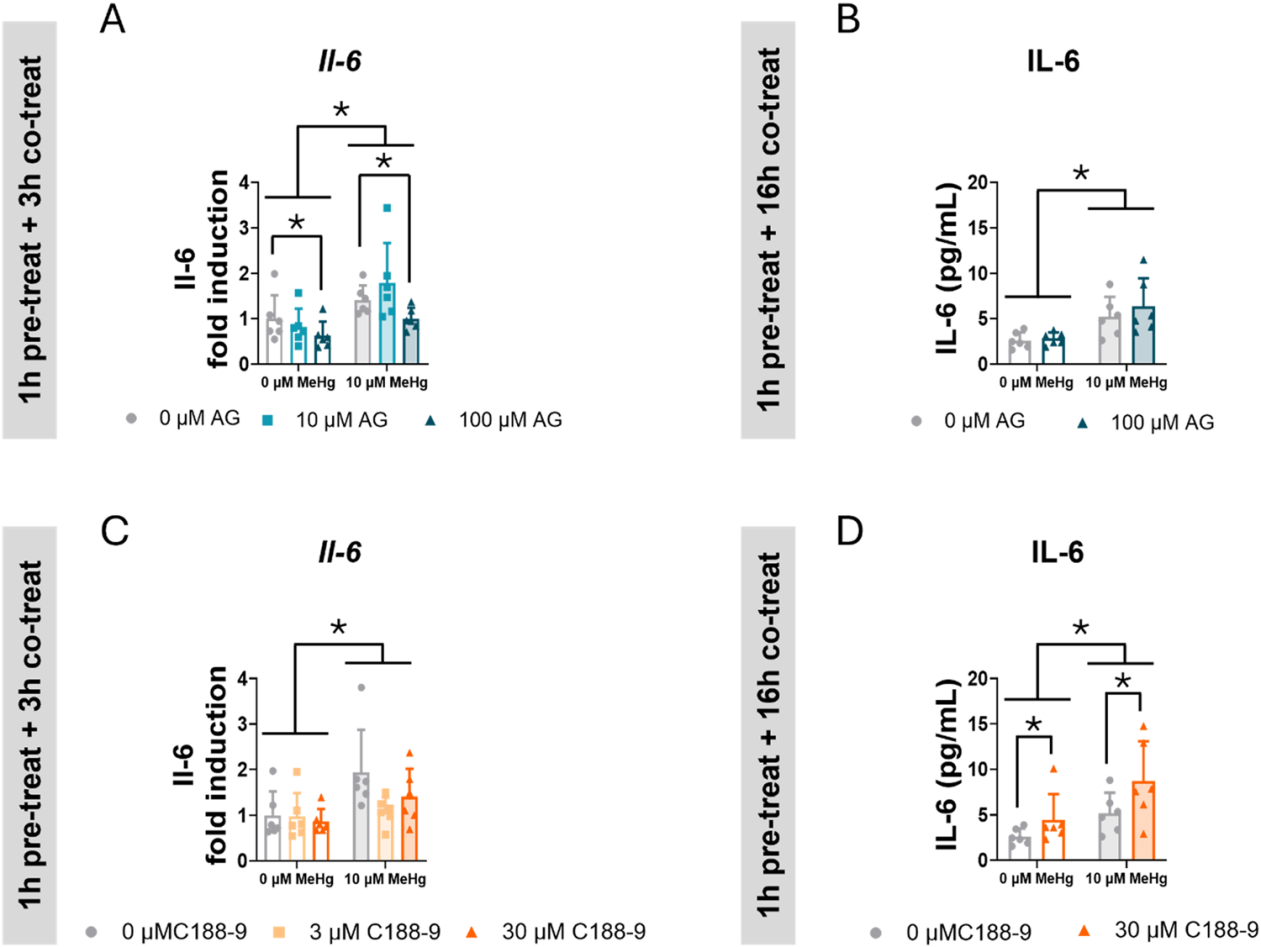
STAT3 inhibition did not prevent MeHg IL-6 release in C8-D1A astrocytic cells. Cells were pretreated with 0, 10 or 100 µM AG490 (**A**, **B**) or with 0, 3, or 30 µM C188-9 (**C**, **D**), followed by co-treatment with 0 or 10 µM MeHg. *Il-*6 mRNA expression (**A**, **C**) was measured by qPCR at 3 h after adding MeHg. IL-6 release into the medium (**B**, **D**) was measured at 16 h after adding MeHg Data are presented as mean ± SD. Statistical significance was determined using two-way ANOVA followed by Bonferroni’s *post-hoc* analysis. When data did not meet the assumptions of normality, a logarithmic or square root transformation was employed before conducting the two-way ANOVA. *p* < 0.05 was considered statistically significant. * denotes a significant difference

MeHg significantly induced *Il-6* mRNA expression in cells pre-treated 1 h with AG490 and co-exposed with MeHg for 3 h (F_MeHg(1,30)_ = 20.547, *p* < 0.001) (Fig. 11A). Conversely, the highest concentration of AG490 (100 µM) decreased *Il-6* mRNA expression (F_AG(2,30)_ = 4.318, *p* = 0.022; *post hoc* *p* = 0.045 compared to non-exposed cells) (Fig. 11A). However, this decrease in the mRNA expression did not correlate with decreased IL-6 release into the medium after 16 h of co-exposure (Fig. 11B). Notably, MeHg significantly increased IL-6 release in these cells (F_MeHg(1,20)_ = 20.692, *p* < 0.001) (Fig. 11B).

In cells pre-treated with C188-9 and co-exposed to MeHg, the toxic compound significantly induced *Il-6* gene expression (F_MeHg(1,30)_ = 10.702, *p* = 0.003) (Fig. 11C), but no effects of C188-9 were observed on it. In these cells, MeHg significantly increased IL-6 release in the medium (F_MeHg(1,20)_ = 11.957, *p* = 0.002) (Fig. 11D). However, after 16 h, C188-9 induced a significant increase in IL-6 release (F_C188(1,20)_ = 5.789, *p* = 0.026) (Fig. 11D).

While MeHg consistently enhances IL-6 expression and release, the role of STAT3 modulating IL-6 appears to be more complex.

### N-acetylcysteine Protected C8-D1A Astrocytic Cells from MeHg-induced Oxidative Stress, Partially Inhibiting STAT3 Phosphorylation

In addition to classical kinase activation, STAT3 is also regulated by oxidative stress [52]. Therefore, MeHg may directly mediate STAT3 activation by binding to STAT3 cysteines, or indirectly by increasing ROS levels. To better understand how MeHg induced STAT3 activation, we inhibited MeHg-induced oxidative stress using two common antioxidants, NAC and Trolox [53].

**Fig. 12:**
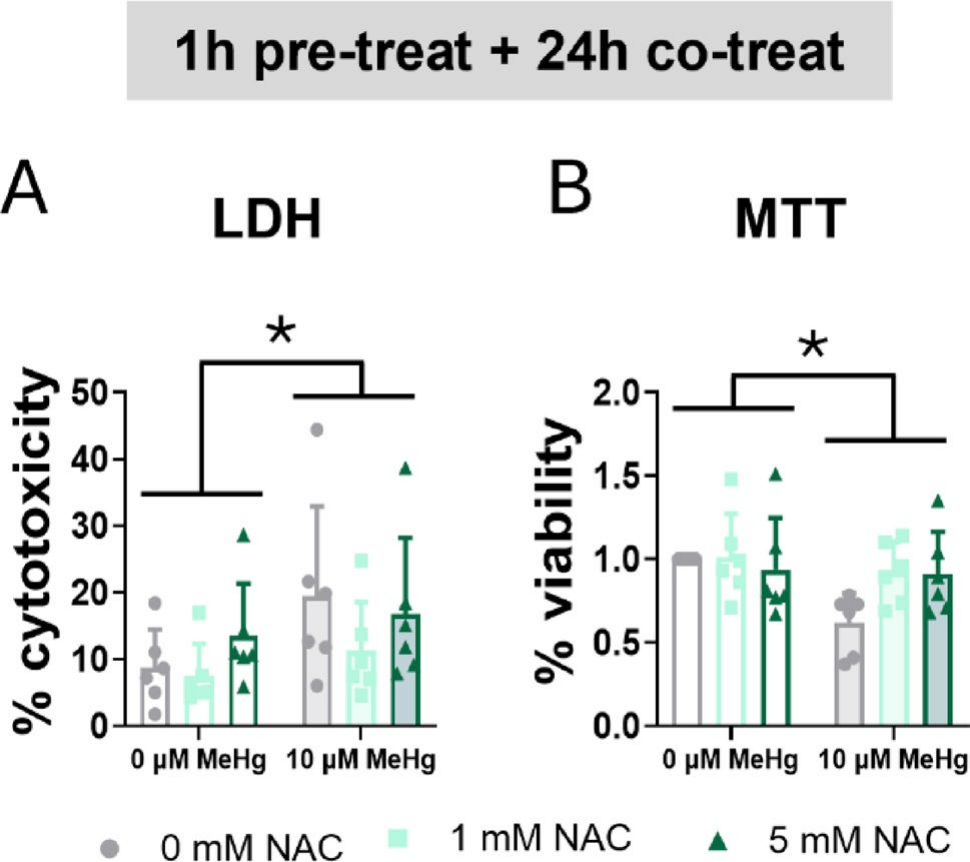
N-acetylcysteine failed to counteract MeHg-induced cytotoxicity. Cells were pretreated with 0, 1, or 5 mM NAC, followed by co-treatment with 0 or 10 µM MeHg. (**A**) Cytotoxicity was measured using LDH assay after 24 h of MeHg addition. (**B**) Cell viability was assessed by MTT assay after 24 h of MeHg addition. Data are presented as mean ± SD. Statistical significance was determined using two-way ANOVA followed by Bonferroni’s *post-hoc* analysis. When data did not meet the assumptions of normality, a logarithmic or square root transformation was employed before conducting the two-way ANOVA. *p* < 0.05 was considered statistically significant. * denotes a significant difference

First, we studied the effects of NAC in cell survival. MeHg significantly induced cytotoxicity (F_MeHg(1,30)_ = 5.113, *p* = 0.031) (Fig. 12A) and decreased cell viability (F_MeHg(1,30)_ = 5.036, *p* = 0.032) (Fig. 12B) at 24 h. However, NAC did not affect cell survival under these conditions.

**Fig. 13:**
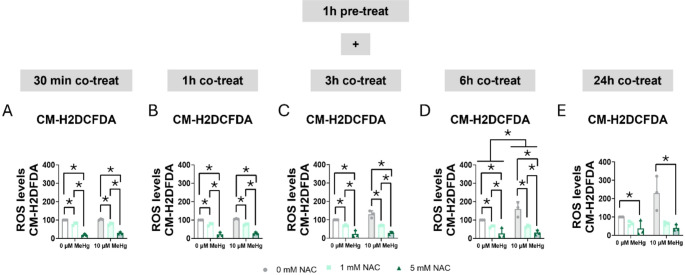
N-acetylcysteine reduced MeHg-induced ROS production. Cells were pretreated with 0, 1, or 5 mM NAC, followed by co-treatment with 0 or 10 µM MeHg. ROS levels were measured using CM-H_2_DCFDA probe at 30 min (**A**), 1 h (**B**), 3 h (**C**), 6 h (**D**), and 24 h (**E**) after MeHg addition. Data are presented as mean ± SD. Statistical significance was determined using two-way ANOVA followed by Bonferroni’s *post-hoc* analysis. When data did not meet the assumptions of normality, a logarithmic or square root transformation was employed before conducting the two-way ANOVA. *p* < 0.05 was considered statistically significant. * denotes a significant difference

Second, we measured the antioxidant effect of NAC on MeHg-induced ROS levels. MeHg increased ROS production, reaching significance at 3 h (F_MeHg(1,12)_ = 4.789, *p* = 0.049) (Fig. 13C), whereas NAC significantly decreased ROS production at 30 min (F_NAC(2,12)_ = 290.735, *p* < 0.001) (Fig. 13A), 1 h (F_NAC(2,12)_ = 229.490, *p* < 0.001) (Fig. 13B), 3 h (F_NAC(2,12)_ = 90.950, *p* < 0.001) (Fig. 13C), 6 h (F_NAC(2,12)_ = 34.811, *p* < 0.001) (Fig. 13D), and at 24 h (F_NAC(2,12)_ = 12.364, *p* = 0.001) (Fig. 13E).

**Fig. 14:**
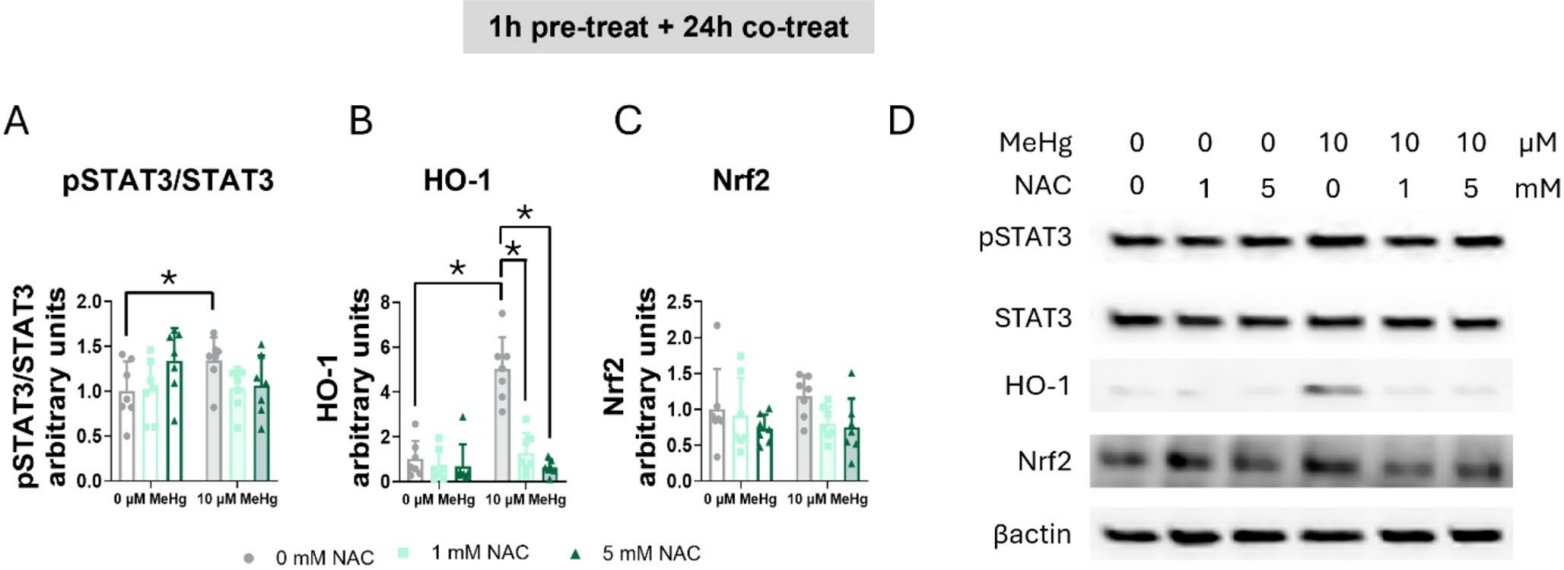
MeHg-induced STAT3 phosphorylation was partially mediated by ROS. Cells were pretreated with 0, 1, or 5 mM NAC, followed by co-treatment with 0 or 10 µM MeHg. (**A**) phosphorylated STAT3 to total STAT3 ratio, (**B**) HO-1, and (**C**) Nrf2 protein levels were measured by western blot 24 h after MeHg addition. (**D**) shows representative densitometry images. Data are presented as mean ± SD. Statistical significance was determined using two-way ANOVA followed by Bonferroni’s *post-hoc* analysis. When data did not meet the assumptions of normality, a logarithmic or square root transformation was employed before conducting the two-way ANOVA. *p* < 0.05 was considered statistically significant. * denotes a significant difference

Finaly, we assessed whether NAC reversed MeHg-induced STAT3 phosphorylation. A significant interaction was found in the phosphorylated STAT3 to total STAT3 ratio (F_IntN(2,36)_ = 3.457, *p* = 0.042) (Fig. 14A, D). Whereas MeHg significantly increased the phosphorylated STAT3 to total STAT3 ratio (*post hoc* *p* = 0048 compared to non-exposed cells), NAC treatment partially blocked this induction (Fig. 14A, D).

To confirm if the antioxidant effects of NAC were mediated by antioxidant defense induction, we measured HO-1 protein levels at 24 h of co-exposure. A significant interaction between MeHg and NAC was found (F_IntN(2,36)_ = 3.614, *p* = 0.037) (Fig. 14B, D). MeHg significantly increased HO-1 protein levels (*post hoc* *p* < 0.001 compared to non-exposed cells), which were restored in cells treated with NAC (for 1 mM NAC *post hoc* *p* < 0.001 compared to MeHg and for 5 mM NAC *post hoc* *p* < 0.001 compared to MeHg). In contrast, no effects of MeHg nor NAC were detected in Nrf2 protein levels at 24 h (Fig. 14C, D).

To further understand the effects of ROS on MeHg-induced STAT3 regulation, we also used Trolox as an antioxidant. Similarly, at 24 h, MeHg significantly induced cytotoxicity (F_MeHg(1,30)_ = 23.942, *p* < 0.001) (Sup. Figure 6 A) and decreased cell viability (F_MeHg(1,30)_ = 50.225, *p* < 0.001) (Sup. Figure 6B). As observed in NAC-treated cells, Trolox did not restore cell survival (Sup. Figure 6 A, B). MeHg significantly elevated ROS levels at 3 h (F_MeHg(1,12)_ = 8.747, *p* = 0.012) (Sup. Figure 7 C), 6 h (F_MeHg(1,12)_ = 10.650, *p* = 0.007) (Sup. Figure 7D), and 24 h (F_MeHg(1,12)_ = 19.067, *p* < 0.001) (Sup. Figure 7E). Unlike NAC, Trolox’s effects on ROS production were less evident; only the low concentration of Trolox (25 µM) significantly decreased ROS levels at 30 min (F_Tx(2,12)_ = 11.427, *p* = 0.002; *post hoc* *p* = 0.001 compared to non-exposed cells) (Sup. Figure 7 A), 1 h (F_Tx(2,12)_ = 11.211, *p* = 0.002; *post hoc* *p* = 0.001 compared to non-exposed cells) (Sup. Figure 7B), and 3 h (F_Tx(2,12)_ = 5.050, *p* = 0.026; *post hoc* *p* = 0.027 compared to non-exposed cells) (Sup. Figure 7 C).

Neither MeHg nor Trolox had any significant effect at 24 h on phosphorylated STAT3 to total STAT3 ratio (Sup. Figure 8 A, D) or Nrf2 protein levels (Sup. Figure 8 C, D). Whereas MeHg significantly increased HO-1 protein expression (F_MeHg(1,36)_ = 185.363, *p* < 0.001), Trolox failed to modulate its expression (Sup. Figure 8 C, D). Our data suggests that under our conditions, Trolox was less efficient than NAC, counteracting MeHg-induced oxidative stress.

## Discussion

The signaling pathways involved in the deleterious effects of MeHg toxicity are not fully characterized. This novel study explores the role of the STAT3 signaling pathway in regulating oxidative stress induced by MeHg. Here, we demonstrate that acute/short term (≤ 24 h) MeHg induced STAT3 activation. Additionally, we show that inhibiting the JAK2/STAT3 pathway exacerbated acute MeHg-induced oxidative stress, leading to enhanced activation of antioxidant defenses in C8-D1A astrocytic cells. Furthermore, MeHg-induced STAT3 phosphorylation is partially mediated by the increase in ROS production.

MeHg has been shown in numerous studies to modify protein phosphorylation in a concentration- and cell-type-dependent manner, resulting in the dysregulation of signaling pathways [54–56]. However, very little is known about the effects of MeHg on the STAT3 signaling pathway. Tan et al. found that ERK/MAPKs and STAT3 signaling pathways may be related to the hormesis of MeHg-HSA in N9 microglial cells [20], while Jebbett and colleagues found that specific levels of MeHg enhanced CNTF-evoked STAT3 phosphorylation, target-gene expression, and glial differentiation in mouse cortical neural progenitor cells [18]. For the first time, the role of STAT3 in MeHg toxicity was implicated in hypothalamic neuronal cells, where MeHg induced STAT3 phosphorylation in a time- and concentration-dependent manner, exacerbating oxidative stress [21]. To understand whether the observed STAT3 functions in hypothalamic neuronal cells are specific to neuronal cells or represent a conserved mechanism in other cell types, we aimed to investigate the role of STAT3 in MeHg-induced toxicity in an astrocytic cell model.

Consistent with previous findings, MeHg induced a time- and concentration-dependent increase in ROS production in C8-D1A astrocytic cells. Our results align with numerous studies reporting that MeHg increases ROS production in astrocytic cells, leading to cellular death [11, 29, 57–60]. It is known that MeHg activates Nrf2 signaling pathway, upregulating antioxidant genes such as HO-1, NQO1, and SLC7A11 in various cell types, including astrocytes, microglia, and neuronal cells [11, 29, 40, 42, 58]. MeHg increased Nrf2 protein levels in a concentration- and time- dependent manner in C8-D1A astrocytic cells. Following MeHg exposure, we also observed an upregulation of antioxidant genes such as *Hmox1* and *Nqo1*, further confirming activation of Nrf2 signaling pathway by MeHg. Interestingly, MeHg also induced a concentration- and time-dependent increase in STAT3 phosphorylation, and in its downstream target genes, *Stat3* and *Socs3*, suggesting that MeHg mediates the activation of STAT3 signaling pathway. Research on the effects of MeHg on STAT3 is limited. Although some studies have suggested that MeHg induced STAT3 phosphorylation in vitro in N9 microglial, SH-SY5Y neuroblastoma, mouse cortical neuronal progenitor, and in GT1-7 hypothalamic neuronal cells [18, 20, 21], and in vivo in the brain of Wistar rats and the hypothalami of C57BL/6 mice [19, 24], its role in MeHg toxicity is not fully understood.

In this study, we found that pharmacological inhibition of STAT3 led to a significant decrease in cell viability in MeHg-exposed cells. This effect was particularly pronounced when STAT3 was inhibited using 30 µM C188-9, which targets its SH2 domain and prevents its dimerization and nuclear translocation [46]. Interestingly, C188-9 exhibited a biphasic pattern depending on the concentration and treatment duration. At 3 h, the lower concentration used (3 µM C188-9) suggested a transient, potentially protective effect against cell death. However, this initial efficacy was not sustained. In contrast, the higher concentration of C188-9 increased LDH release at 6 and 24 h, indicating cell membrane damage. This was confirmed by decreased MTT reduction at 24 h, demonstrating a clear cytotoxic effect over time.

STAT3 has been shown to play a neuroprotective role against glutamate-induced excitotoxicity in primary cultures of dorsal root ganglion neurons and glioblastoma cells [61, 62]. Activated STAT3 promotes cell survival and proliferation through the upregulation of Cyclin D1, PIM-1, and BCL-2 [63–65]. Interestingly, several studies have suggested that MeHg interferes with cell cycle progression, leading to cell death [66–68]. MeHg exposure regulated Cyclin D1 in a concentration-dependent manner, with distinct patterns observed in different brain regions. Higher MeHg concentrations increased Cyclin D1, whereas lower concentrations decreased it [69]. Notably, MeHg reduced Cyclin D1 expression in hippocampus, while in cerebellum no effects were observed [66]. The effects of MeHg on Cyclin D1 may involve the regulation of NF-κ B and STAT3 signaling pathways. We previously reported that in GT1-7 hypothalamic cells, MeHg-induced mortality correlated with the decrease of *Ccnd1* and *Pim1* mRNA after inhibition of JAK2/STAT3 [21].

The inhibition of STAT3 in GT1-7 hypothalamic neuronal cells exacerbated oxidative stress and increased antioxidant genes, suggesting a possible role in antioxidant defense [21]. Notably, when STAT3 activity was inhibited by AG490, which targets its upstream kinase JAK2, ROS production was exacerbated. These findings align with a previous study indicating that disruption of astrocyte STAT3 signaling decreases mitochondrial function and increases oxidative stress in vitro [70]. Surprisingly, this effect was not observed when STAT3 activity was prevented by blocking SH2 domain dimerization using C188-9, which partially ameliorated MeHg-induced superoxide production. The reasons for these discrepancies are unclear. Studies have shown that STAT3 could be localized in the mitochondria [71–73]. STAT3 mitochondrial localization has been linked to phosphorylation of the serine 727 residue of STAT3 [71, 72]. Blocking the STAT3 SH2 domain is linked with a decrease in STAT3 phosphorylation at the serine 727 residue and impaired mitochondrial function [74]. In PC12 cells, a serine dominant negative mutant of STAT3 reduced nerve growth factor-induced ROS production [75], suggesting a role for serine-phosphorylated STAT3 in mitochondrial ROS production. Therefore, the absence of C188-9 effects on MeHg-induced ROS production observed in our cells, may be mediated by inhibition of serine phosphorylation of STAT3 and a failure in translocation into the mitochondria. Further experiments measuring serine phosphorylation and STAT3 mitochondrial localization may help to clarify these results.

During oxidative stress conditions, Nrf2 translocates into the cell nucleus, forms a dimer with sMaf, and binds to antioxidant response elements (AREs) located in the regulatory regions of genes responsible for cellular defense [41]. Numerous studies have revealed an indirect relationship between STAT3 and Nrf2, wherein decreased STAT3 phosphorylation results in increased Nrf2 expression [76]. The relationship between STAT3 and Nrf2 is also supported by computational analysis of the connection between Nrf2 and STAT1 and STAT3 [77]. As expected, HO-1 levels were increased in MeHg-exposed cells compared to the control, suggesting the activation of the Nrf2 signaling pathway. This finding is consistent with the established role of Nrf2 in mediating cellular resistance and protecting against toxicity [78]. Furthermore, activation of the Nrf2/HO-1 signaling pathway has been shown to inhibit excessive oxidative stress and inflammation [23, 79]. In this study, inhibition of STAT3 pathway enhanced the MeHg-induced expression of antioxidant enzymes, especially HO-1. Direct inhibition of STAT3 with C188-9 further enhanced MeHg-induced *Hmox1* gene expression, suggesting a role for STAT3 in regulating antioxidant stress defense.

Another key antioxidant defense mechanism against MeHg is GSH. In vivo models have shown that developmental exposure to MeHg decreases GSH brain levels [39, 80]. Similar results have been observed in primary astrocytic cultures [11, 81, 82]. Although MeHg-induced GSH depletion is an expected event, surprisingly, in our cell model, MeHg did not affect total or reduced GSH levels. Similar to our findings, astrocytoma cells have shown resistance to MeHg-induced GSH depletion [83]. Altogether, these results suggest that different cell types may present different vulnerabilities to MeHg. More interestingly, the effects of STAT3 inhibition on GSH levels were observed. AG490 induced GSH depletion, and this effect was exacerbated in cells exposed to MeHg. Therefore, adequate GSH levels are needed to ensure protection against MeHg. GSH depletion leads to enhanced MeHg toxicity [84]. Similar effects of AG490 inhibitor have been shown in other disease models where oxidative stress plays a pathological role. In myocardial ischemia and infarction models, the protective effects of GSH-inducing substances were abolished by AG490 treatment [85–88], suggesting a role for JAK2/STAT3 in modulating GSH levels. Interestingly, direct inhibition of STAT3 via blocking its SH2 domain using the C188-9 inhibitor did not elicit any effect on GSH levels, suggesting that GSH may be regulated by JAK2 rather than by STAT3. It is important to note that the AG490 inhibitor mediates inhibition of STAT3 phosphorylation by targeting its upstream kinase, JAK2. It is known that JAK2 also regulates phosphorylation of other STAT members, such as STAT1, STAT2, or STAT5 [89–91]. Therefore, we cannot rule out that the AG490-induced GSH decrease may be mediated by other STATs.

Although the potential role of inflammation in MeHg-induced neurodegeneration remains to be elucidated, studies suggest that MeHg triggers neuroinflammatory markers [92–94]. In vivo studies have demonstrated that MeHg exposure increased IL-6 serum levels in rodents [95–97]. Consistently, in vitro studies using glial cells have shown that MeHg induces IL-6 at high concentrations [98–102]. In our study, MeHg induced *Il-6* gene expression and release in C8-D1A astrocytic cells, supporting the published data. Studies in different disease models have shown that AG490 treatment counteracts IL-6 induction [103–106], suggesting a role for the JAK2/STAT3 pathway in the regulation of IL-6 expression. AG490 inhibited MeHg-induced *Il-6* gene expression, but it did not affect IL-6 release. Contrary to the AG490, C188-9 did not affect *IL-6* gene expression in C8-D1A astrocytic cells. Similar findings have been reported in kidney and fibroblast cells, where the C188-9 failed to modulate IL-6 expression [107, 108]. Although we did not observe changes in gene expression, the C188-9 increased IL-6 release. The differences observed between both inhibitors suggest that other pathways may be implicated in MeHg-induced IL-6 regulation. Additionally, Wruck et al. (2011) propose an interaction between Nrf2 and IL-6, suggesting that Nrf2 may play a role in inflammatory responses by binding to antioxidant response elements in the promoter region of the *Il-6* gene and activating its transcription [109]. Therefore, a link between antioxidant and anti-inflammatory mechanisms may exist. Further research is needed to investigate the implication of inflammatory genes such as the *IL-6* gene in the defense response against MeHg toxicity.

The nuclear factor kappa B (NF-κB) signaling pathway induces the expression of pro-inflammatory cytokines, such as IL-6 [110, 111]. Notably, STAT3 and NF-κB exhibit crosstalk. They can interact directly or indirectly, regulating biological functions, such as inflammatory processes [112, 113]. STAT3 and NF-κB co-regulate the expression of several genes, including *Socs3*, *Bcl2*, and *Il-6* [112, 114]. This crosstalk is further supported by the physical interaction between STAT3 and p65/p50 NF-κB subunits [115]. Similarly, Nrf2 also interacts with NF-κB, suggesting crosstalk [116]. Both pathways are activated upon exposure to different stressors, such as lipopolysaccharide or cigarette smoke [117, 118]. Several mechanisms regulated the intricate interplay between NF-κB and Nrf2. NF-κB represses Nrf2 transcription through competition for the transcription co-activator CREB (CBP) or by recruiting histone deacetylase 3 to the antioxidant response elements (ARE) sequences [119]. In summary, these interconnected pathways form a sophisticated regulatory core that coordinates both inflammatory and antioxidant responses.

Intracellular ROS are involved in MeHg toxicity [57], playing a pivotal role in the activation of antioxidant defense mechanisms, such as Nrf2 pathway [41, 120]. Similar to Nrf2, STAT3 is phosphorylated in response to oxidative stress [121–123]. NAC, a derivative of the naturally occurring amino acid L-cysteine, and Trolox, a water-soluble vitamin E analog, are two common antioxidants that may mediate protection against MeHg neurotoxicity [53]. Once MeHg reaches the cell, it could induce ROS production, which in turn may activate STAT3. Alternatively, MeHg may directly bind to cysteines present in STAT3, leading to aberrant activation of signaling pathway. NAC and Trolox decreased MeHg-Induced ROS levels in C8-D1A cells; however, Trolox was less efficient than NAC. Consistent with the changes in ROS levels, NAC decreased HO-1 protein expression, and ameliorated MeHg-induced STAT3 phosphorylation. Interestingly, Trolox failed to modulate the expression of these proteins. Trolox’s inability to counteract MeHg-induced HO-1 protein or *Hmox1* mRNA has been previously described in HEK293, HeLa cells and cerebellar primary astrocytic cultures [13, 59]. The differences observed between Trolox and NAC could be explained by the chelating effects of NAC. NAC binds MeHg through its thiol groups, facilitating the reduction of MeHg within the cell by inhibiting the entry of the NAC-MeHg complexes and by promoting MeHg efflux [59, 124]. Interestingly, NAC only partially reversed MeHg-induced phosphorylated to total STAT3 ratio, suggesting that MeHg-induced STAT3 activation is only partly mediated by the increase in ROS production. Therefore, MeHg may mediate STAT3 activation by directly binding to it or indirectly by inactivating the enzymatic activity of upstream inhibitors such as PTP1B. Although NAC was effective in partially reversing MeHg-induced STAT3 phosphorylation and modulating HO-1 expression, these effects may primarily reflect its capacity to chelate MeHg, thereby reducing its bioavailability, rather than a direct antioxidant action. This limitation should be considered when interpreting the redox-related effects observed in this study. Future experiments using mitochondria-targeted ROS scavengers such as Mito-TEMPO will be necessary to clarify these mechanisms.

However, we measured antioxidant effects at 24 h, a time point where the STAT3 phosphorylation had already begun to revert to basal levels. While our aim was to assess the sustained effects of MeHg and antioxidant co-treatment, this timing might not fully capture the STAT3 activation driven by early ROS signaling. Therefore, future studies including earlier time points will be essential to better define the temporal dynamics of ROS-mediated STAT3 activation and its functional consequences.

In this study, we explored the complex relationship between oxidative stress, Nrf2, and JAK2/STAT3 signaling pathways in MeHg neurotoxicity. Our data demonstrated that MeHg induces STAT3 activation, and the dysregulation of this pathway exacerbates MeHg toxicity, suggesting a possible role for STAT3 in mediating its detrimental effects. These effects are similar to those we previously reported in the hypothalamic neuronal GT1-7 cell line, supporting the notion that MeHg-induced STAT3 activation may represent a shared mechanism with neurons. However, whether other CNS cell types, such as microglia, also exhibit this mechanism remains to be explored. Further analysis is needed to achieve a better understanding of these intricate mechanisms.

## Conclusions

In conclusion, the data presented in this study demonstrates the neuroprotective role of STAT3 as a defense response in MeHg-induced toxicity. STAT3 Inhibitors decreased cell proliferation, increased ROS production levels, and induced an aberrant activation of antioxidant defense in MeHg-exposed C8-D1A cells, leading to an exacerbation of MeHg cytotoxicity. Future research is warranted to fully understand the protective role of STAT3 and its relation to the Nrf2-signaling pathway, as well as the expression of antioxidant genes.

## Supplementary Information

Below is the link to the electronic supplementary material.

Supplementary Material 1

Supplementary Material 2

## Data Availability

The data supporting the findings of this study are available from the corresponding author upon reasonable request.
