# Supplementary material for: JAK2/STAT3 Signaling Pathway Modulates Acute Methylmercury Toxicity in the Mouse Astrocyte C8-D1A Cell Line
Source: Neurochem Res. 2025 Aug 13;50(4):265. doi: 10.1007/s11064-025-04507-7 (PMC12350482; doi:10.1007/s11064-025-04507-7)

Fig. 2E

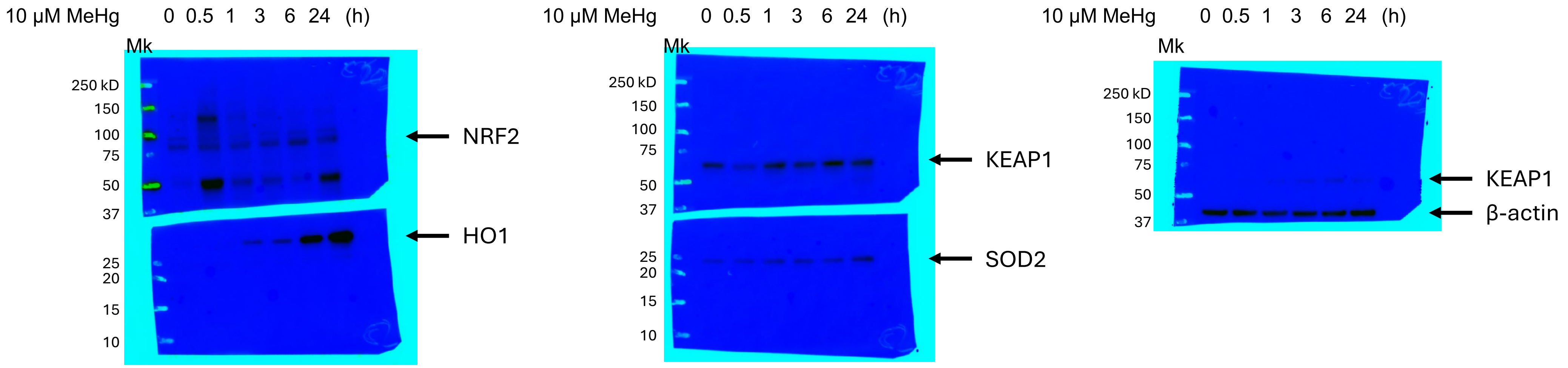
10 μM MeHg 0 0.5 1 3 6 24 (h)
Mk
250 kD
150
100
75
50
37
25
20
15
10
NRF2
HO1
10 μM MeHg 0 0.5 1 3 6 24 (h)
Mk
250 kD
150
100
75
50
37
25
20
15
10
KEAP1
SOD2
10 μM MeHg 0 0.5 1 3 6 24 (h)
Mk
250 kD
150
100
75
50
37
KEAP1
β-actin

Fig. 2E

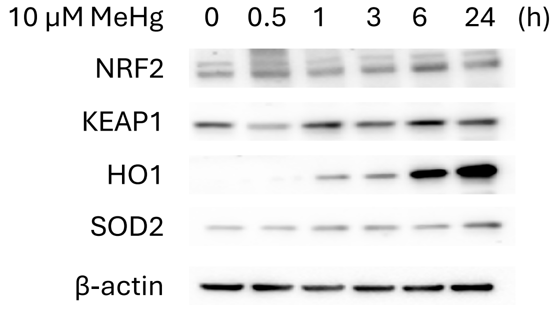
10 μM MeHg 0 0.5 1 3 6 24 (h)
NRF2
KEAP1
HO1
SOD2
β-actin

Fig. 3C

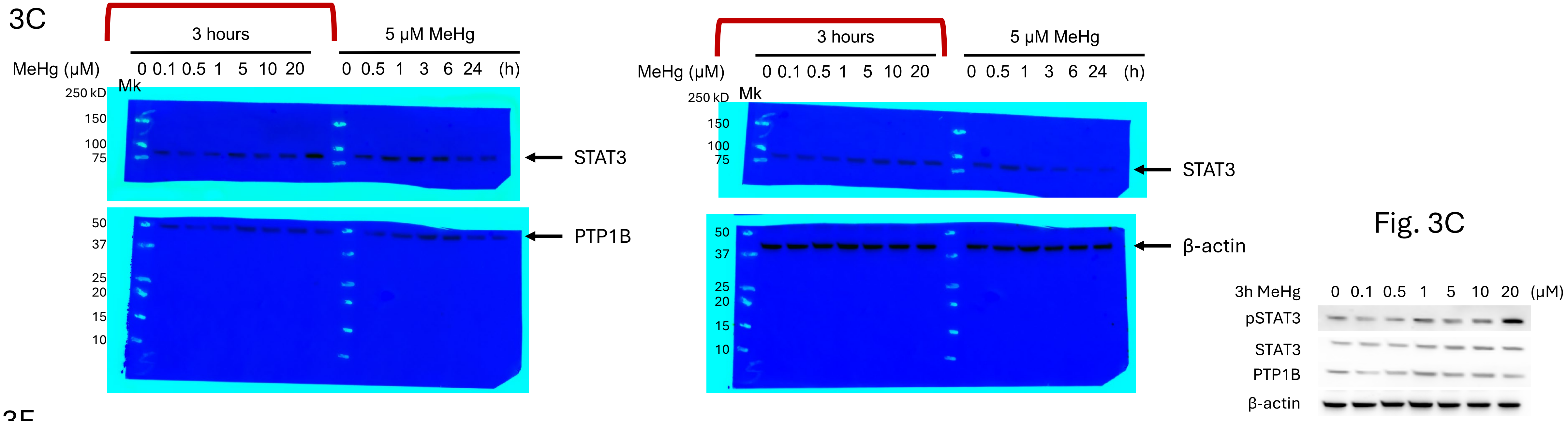
3 hours
5 μM MeHg
MeHg (μM)
0 0.1 0.5 1 5 10 20
0 0.5 1 3 6 24 (h)
Mk
250 kD
150
100
75
50
37
25
20
15
10
STAT3
PTP1B
β-actin
Fig. 3C
3h MeHg
0 0.1 0.5 1 5 10 20 (μM)
pSTAT3
STAT3
PTP1B
β-actin

Fig. 3F

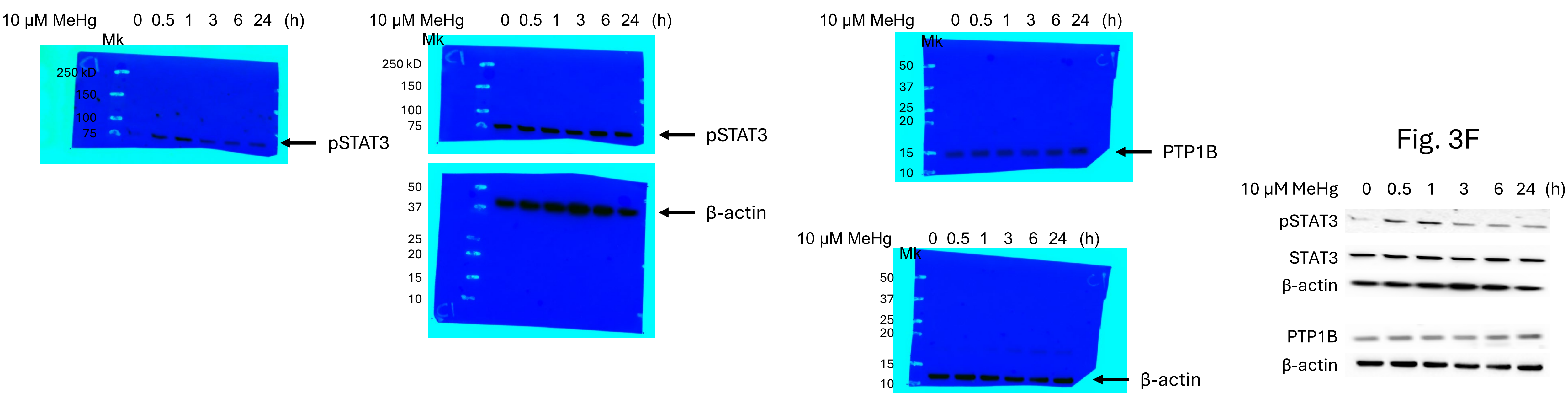
10 μM MeHg
0 0.5 1 3 6 24 (h)
Mk
250 kD
150
100
75
50
37
25
20
15
10
pSTAT3
β-actin
PTP1B
Fig. 3F
pSTAT3
STAT3
β-actin
PTP1B
β-actin

Fig, 9

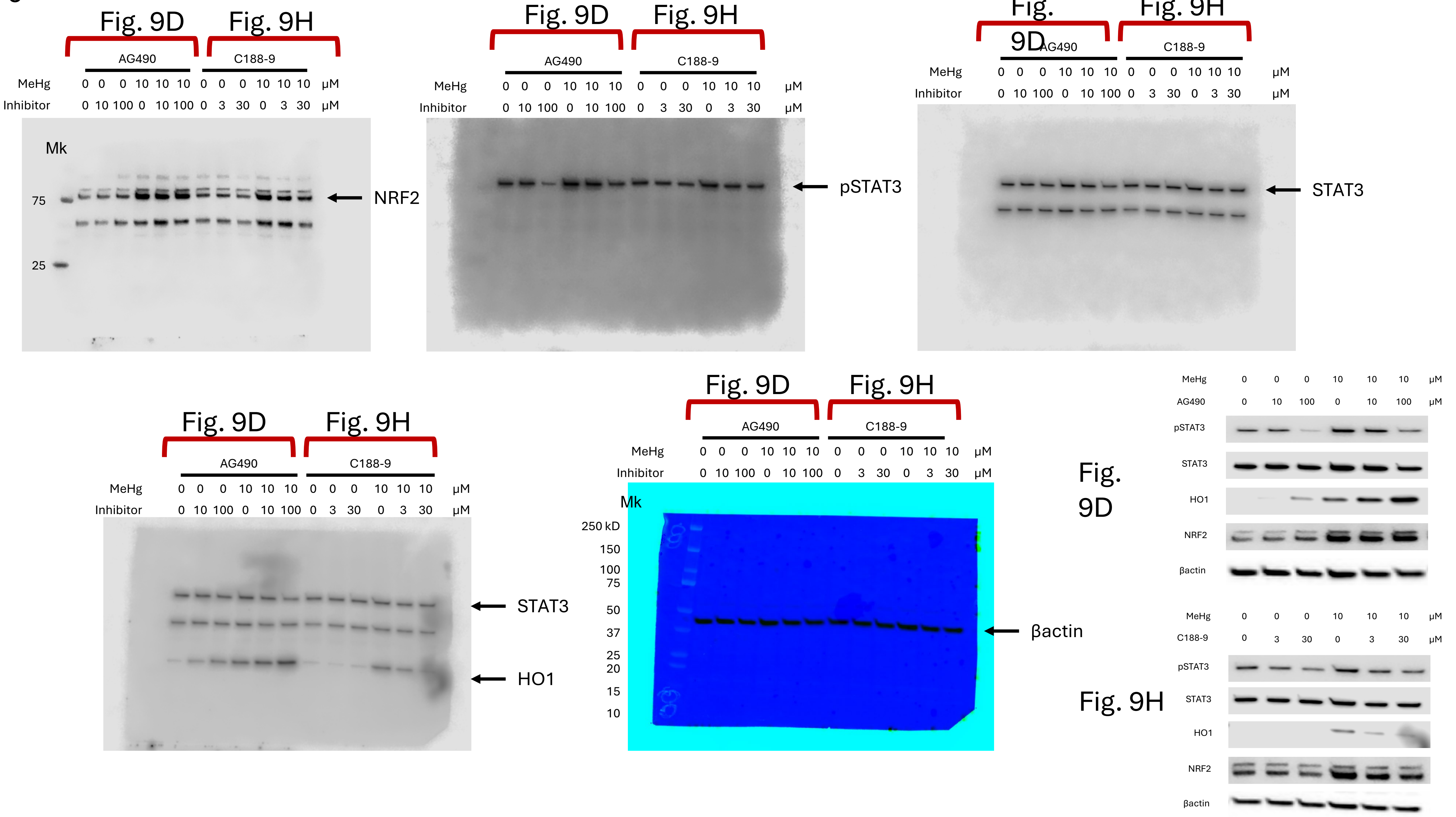
Fig. 9D
Fig. 9H
AG490
C188-9
MeHg 0 0 0 10 10 10 0 0 0 10 10 10 μM
Inhibitor 0 10 100 0 10 100 0 3 30 0 3 30 μM
Mk
75
25
NRF2
pSTAT3
STAT3
HO1
250 kD
150
100
75
50
37
25
20
15
10
βactin
MeHg 0 0 0 10 10 10 μM
AG490 0 10 100 0 10 100 μM
C188-9 0 3 30 0 3 30 μM
pSTAT3
STAT3
HO1
NRF2
βactin

Fig. 14D, and Sup. Fig. 8D

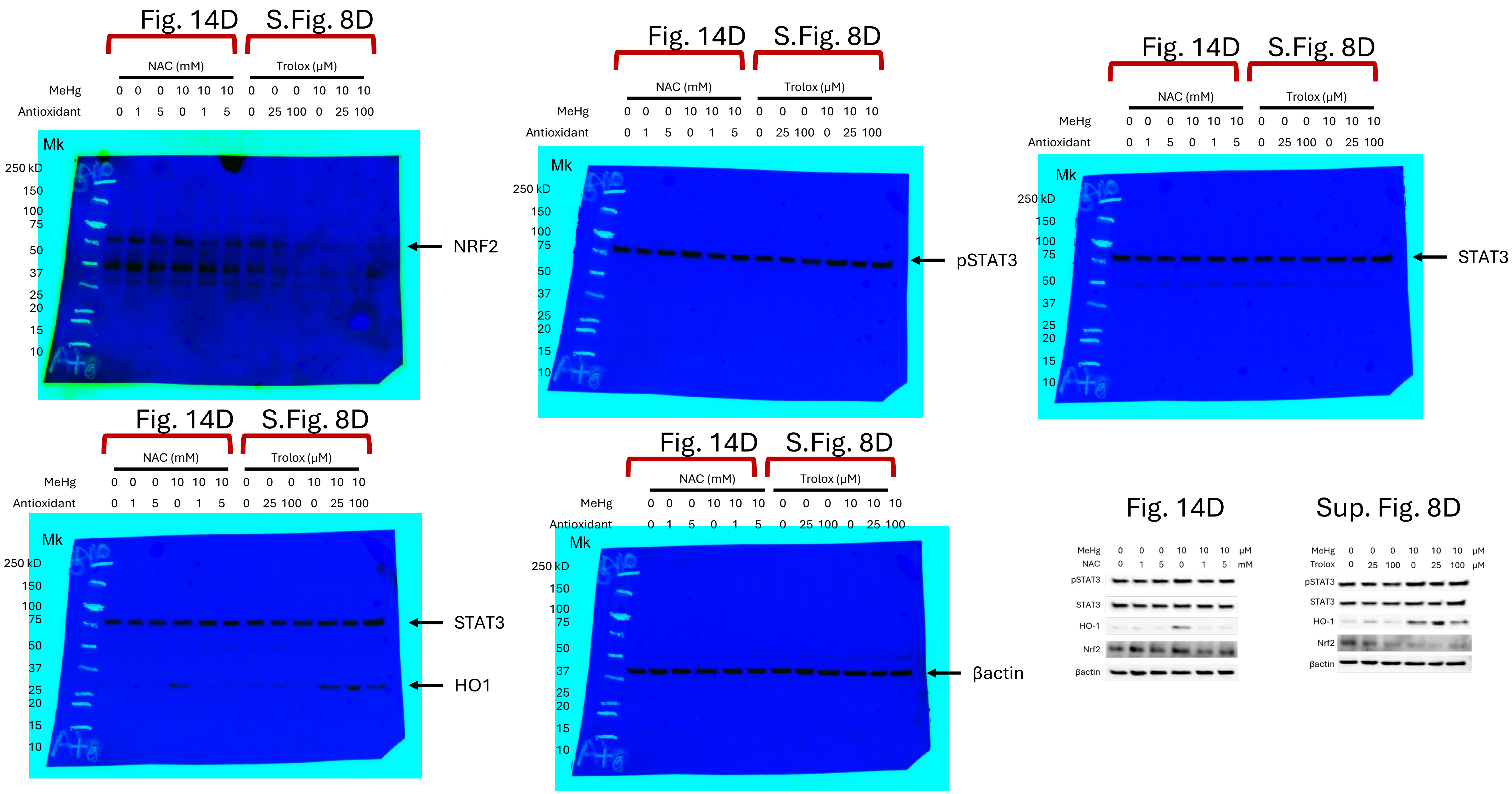

Sup. Fig. 2E

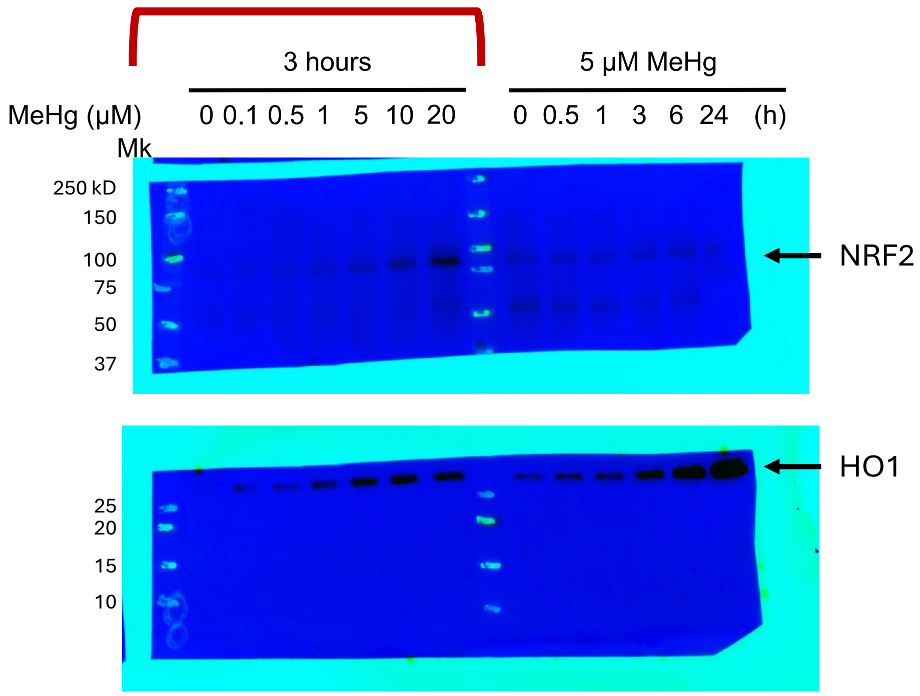

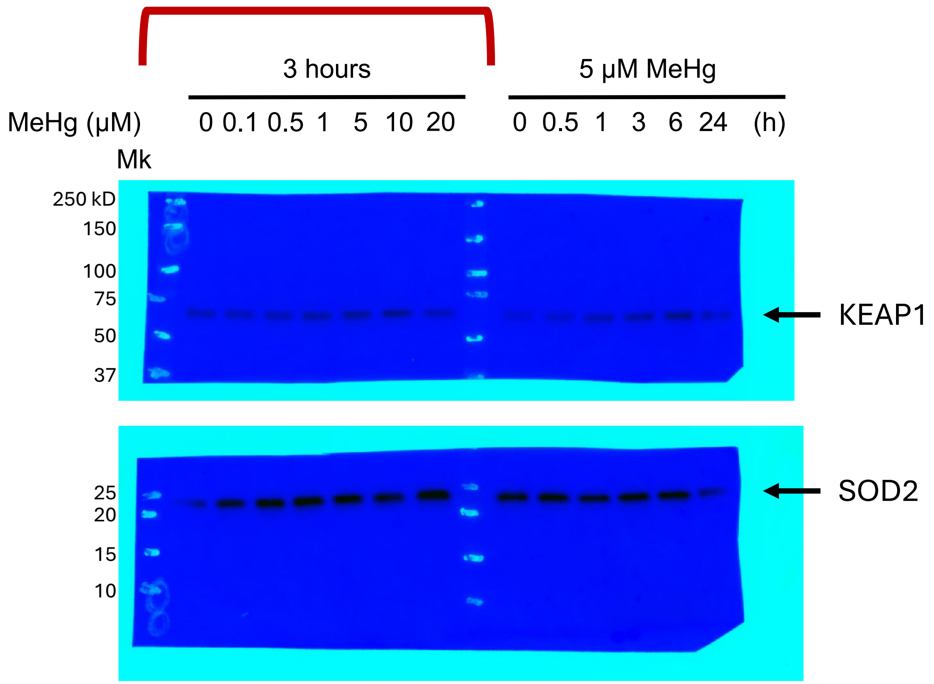

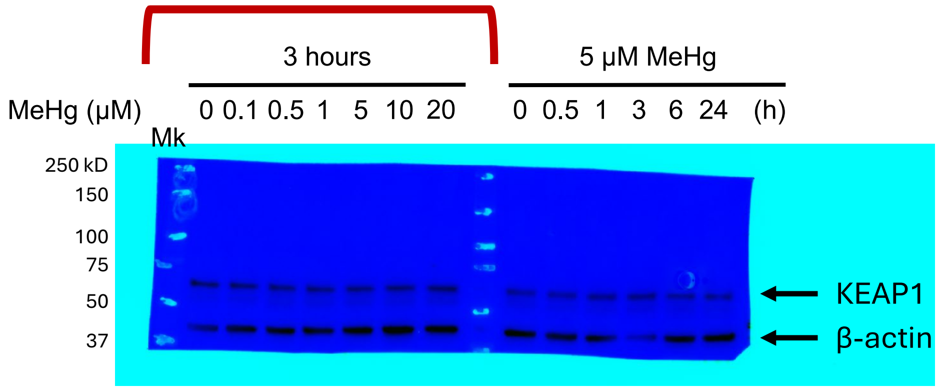

Sup. Fig. 2E

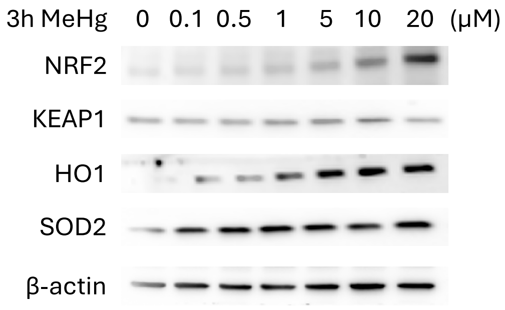

Sup. Fig. 3C

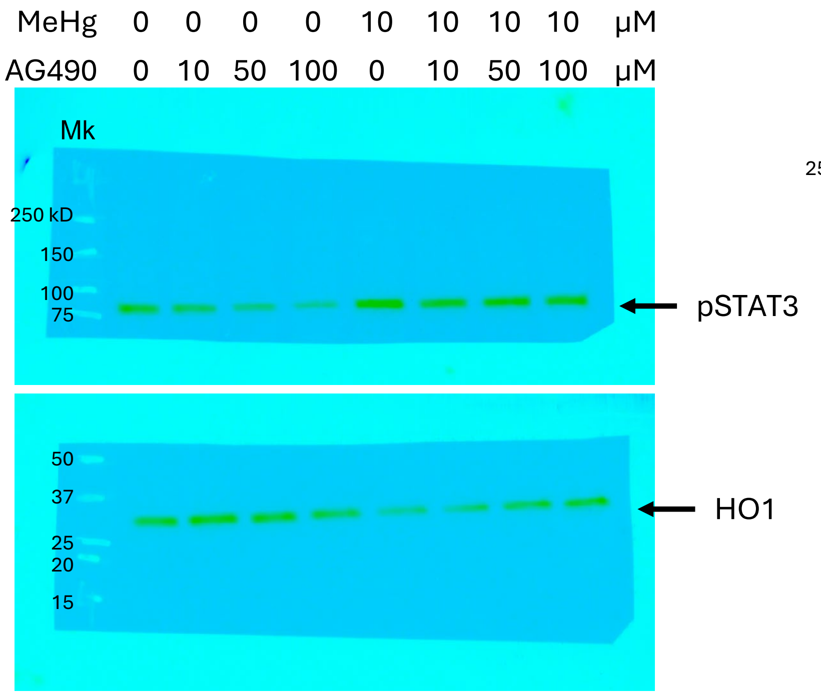

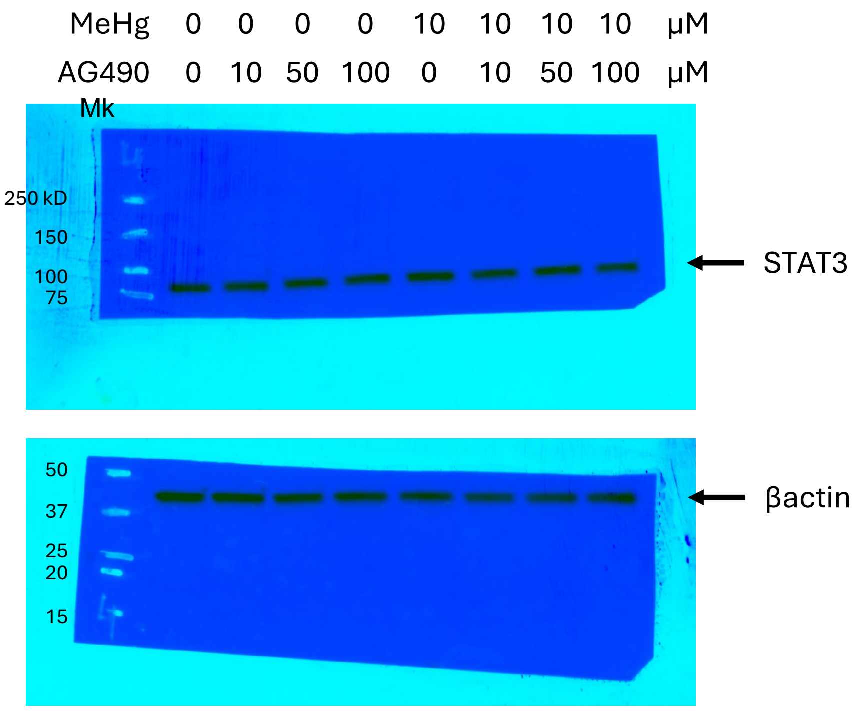

Sup. Fig. 3C

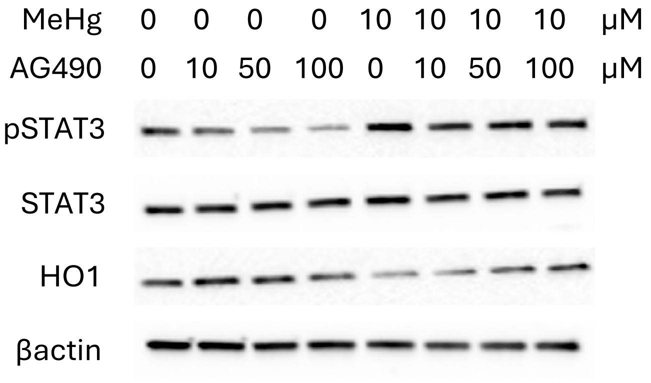

Sup. Fig. 3H

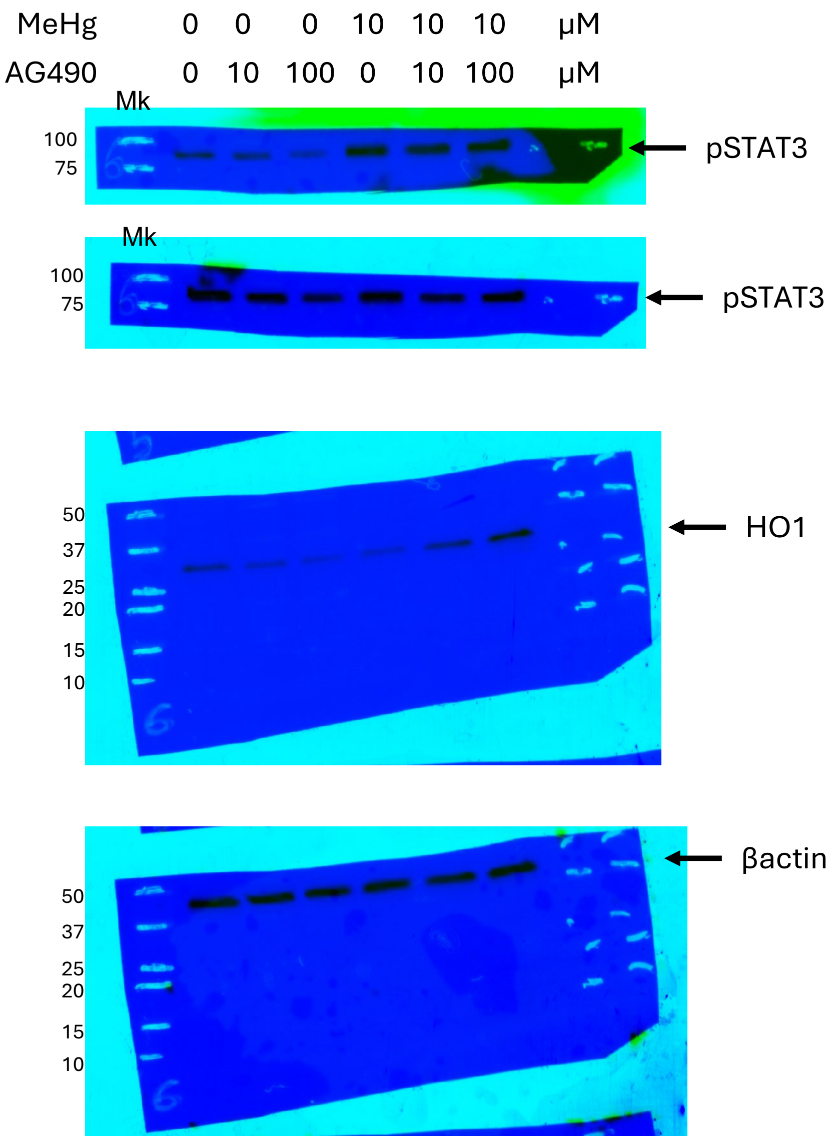

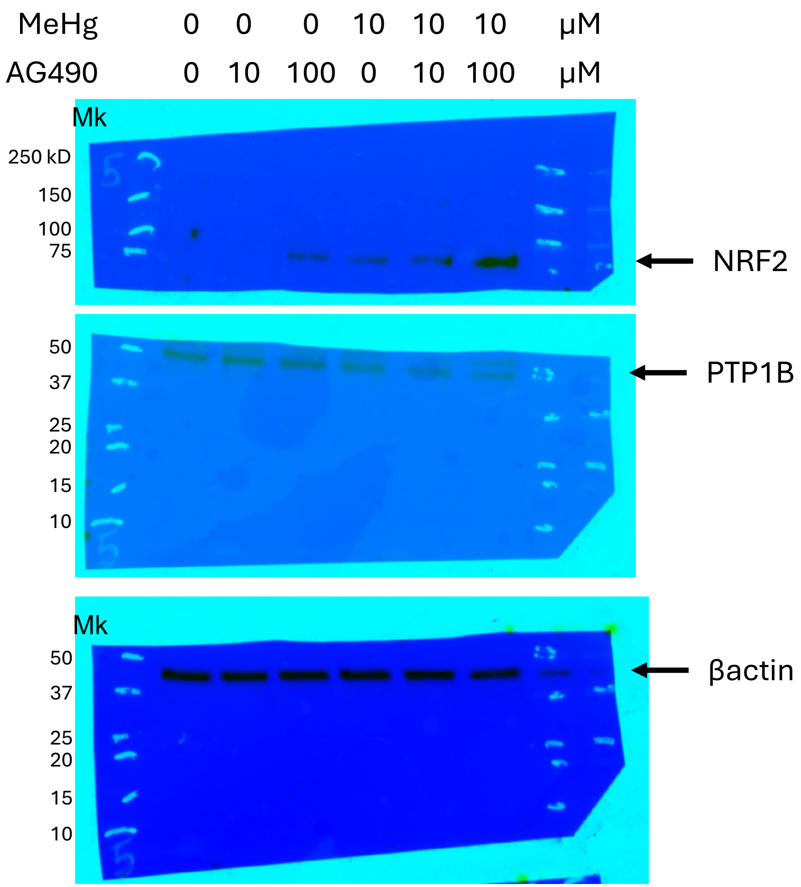

Sup. Fig. 3H

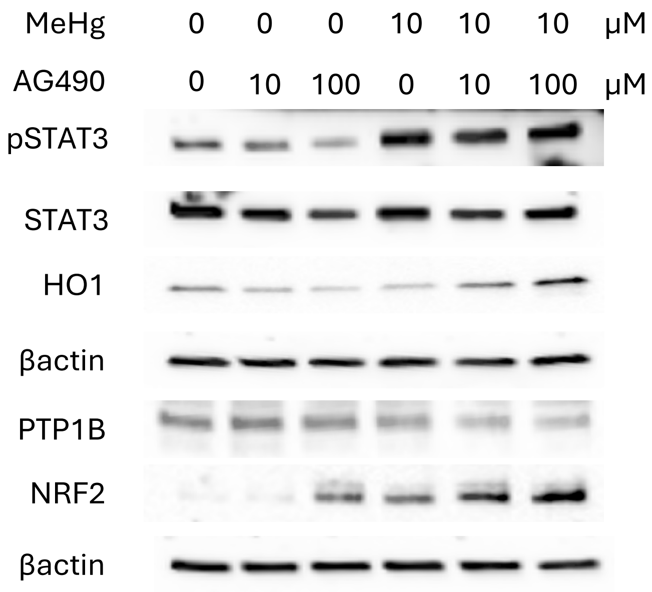

Sup. Fig. 3K and Sup. Fig. 4H

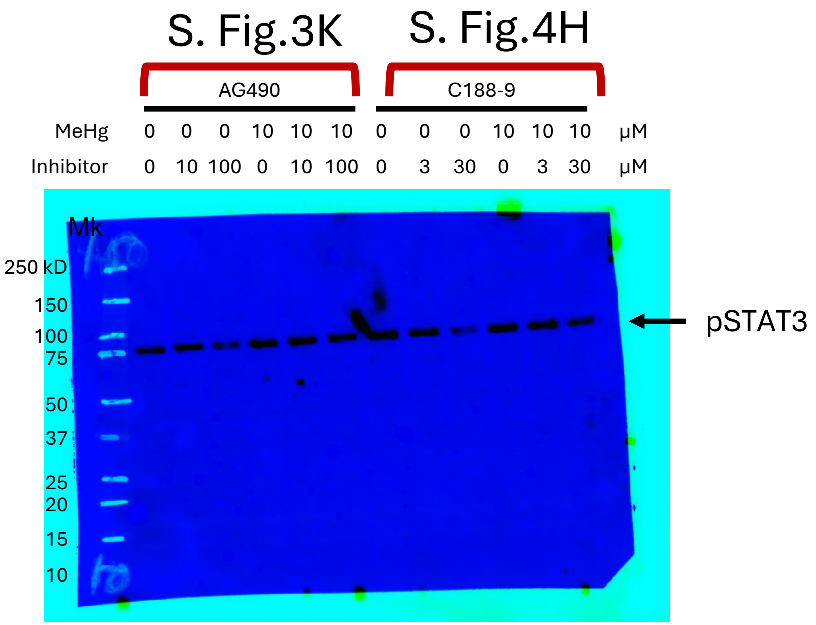

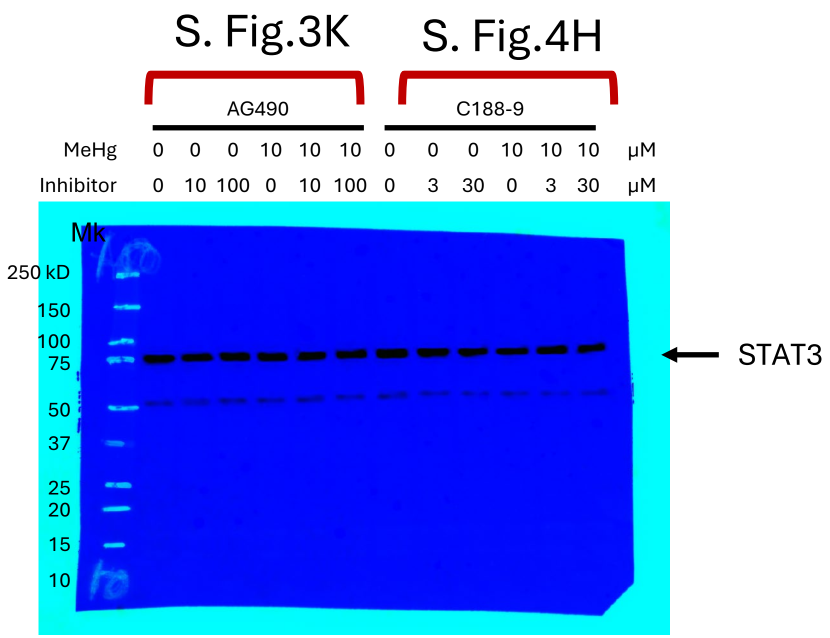

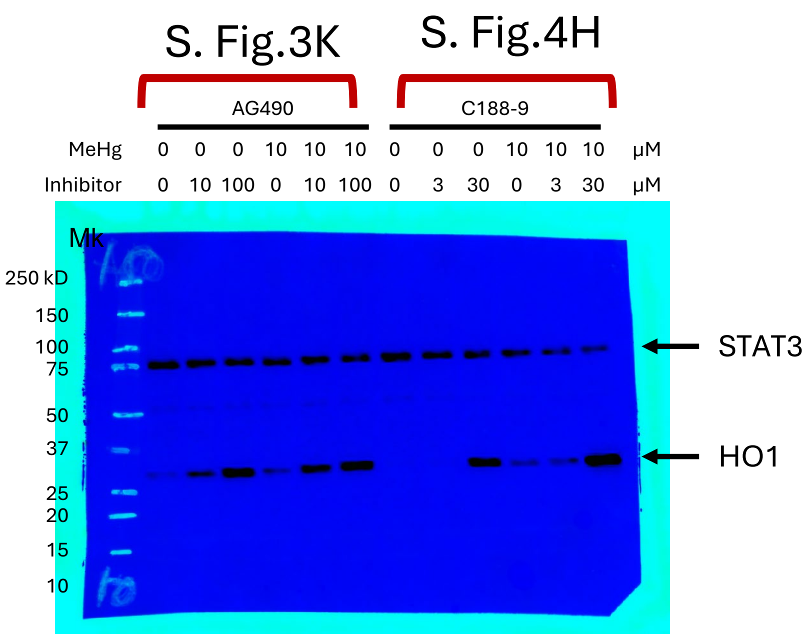

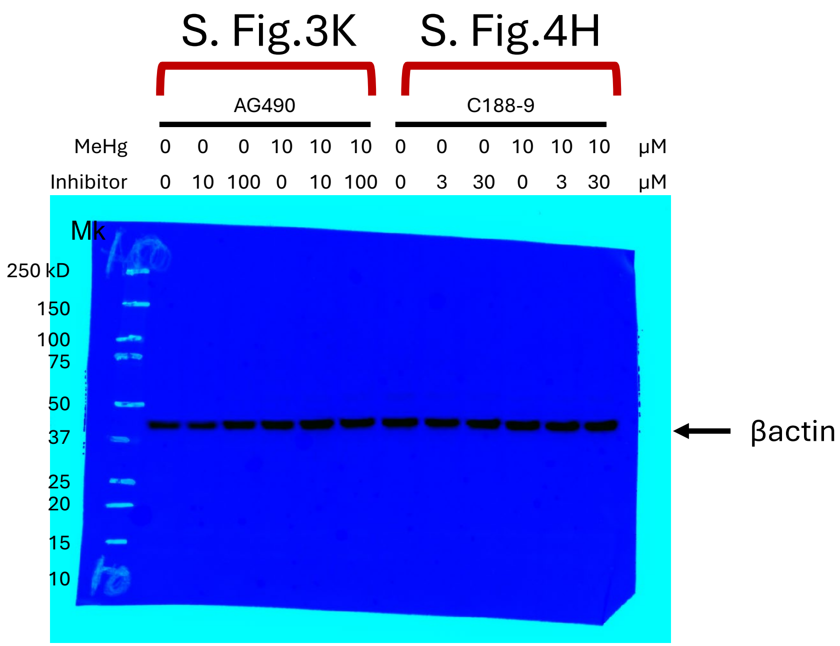

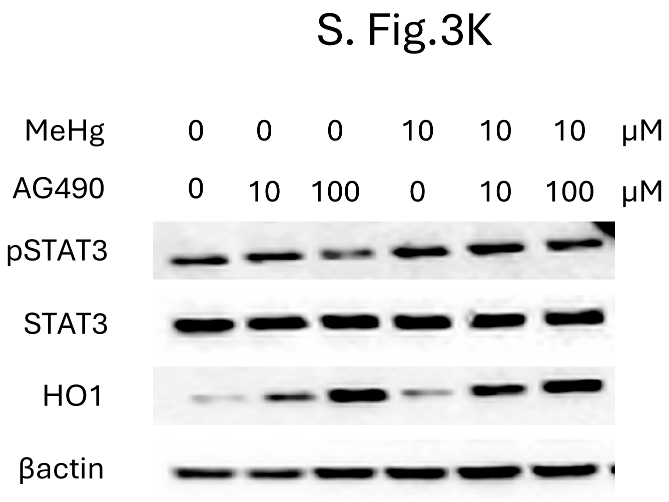

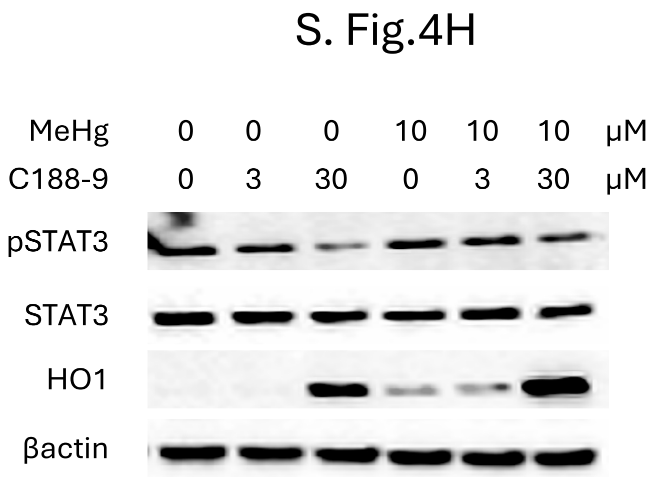

Sup. Fig. 4E

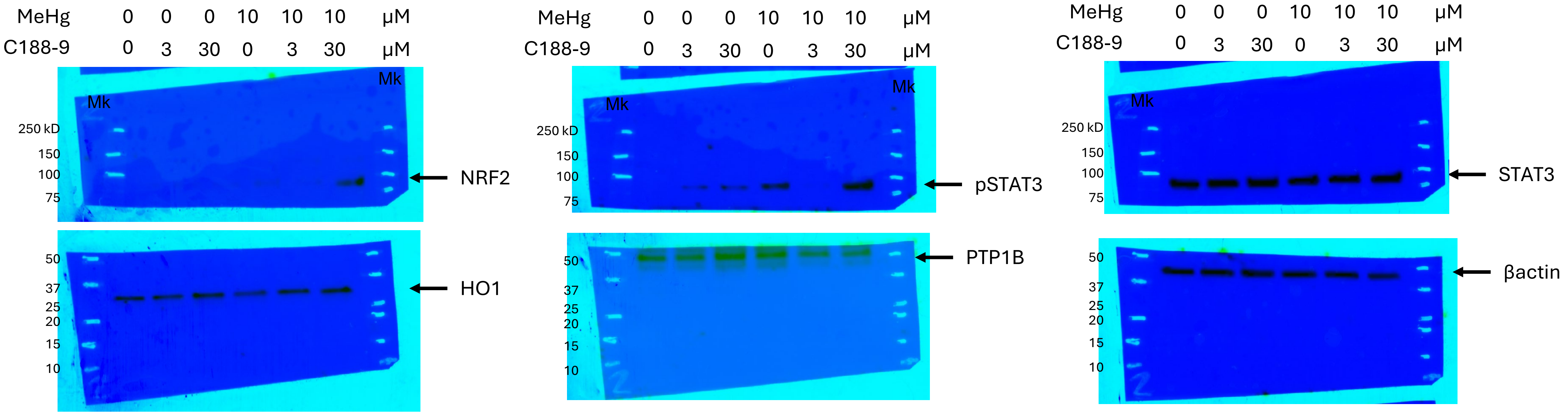

Sup. Fig. 4E

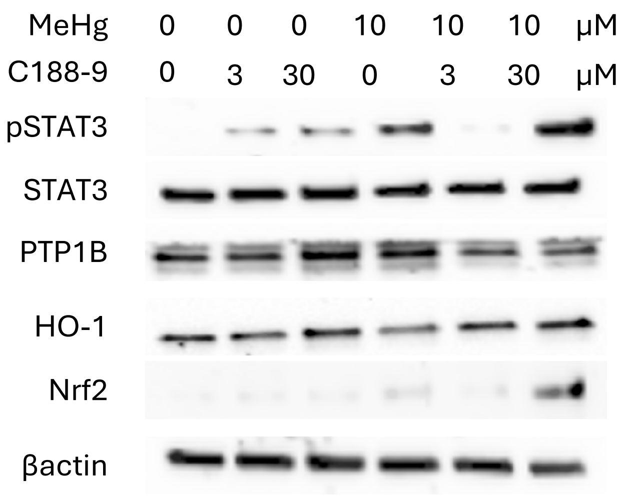

Supplement: Supplementary file 1 — Supplementary Material 1 [file 11064_2025_4507_MOESM1_ESM.pdf]
