# Supplementary material for: JAK2/STAT3 Signaling Pathway Modulates Acute Methylmercury Toxicity in the Mouse Astrocyte C8-D1A Cell Line
Source: Neurochem Res. 2025 Aug 13;50(4):265. doi: 10.1007/s11064-025-04507-7 (PMC12350482; doi:10.1007/s11064-025-04507-7)
Supplement: Supplementary file 2 — Supplementary Material 2 [file 11064_2025_4507_MOESM2_ESM.docx]

SUPPLEMENTARY MATERIALS

**TITLE**

JAK2/STAT3 signaling pathway modulates acute methylmercury toxicity in the mouse astrocyte C8-D1A cell line

**AUTHORS**

Aafia Ahmed^1^ - ahmedaafia52@gmail.com

Maximus Wong^1^ - maxwstuff@gmail.com

Abel Santamaria^2,3^ - absada@yahoo.com

João Batista Rocha^4^- jbtrocha@gmail.com

Aaron B Bowman ^5^- bowma117@purdue.edu

Michael Aschner^1^ - michael.aschner@einsteinmed.edu

Beatriz Ferrer^1@^- [beatriz.ferrervillahoz@einsteinmed.edu](mailto:beatriz.ferrervillahoz@einsteinmed.edu)

^@^Corresponding author:

Albert Einstein College of Medicine; Jack and Pearl Resnick Campus; Department of Molecular Pharmacology, 1300 Morris Park Avenue, Bronx, NY 10461, Forchheimer Building.

1. Department of Molecular Pharmacology, Albert Einstein College of Medicine, 1300 Morris Park Avenue, 10461 Bronx, NY, United States.
2. Laboratorio de Nanotecnología y Nanomedicina, Departamento de Atención a la Salud, Universidad Autónoma Metropolitana-Xochimilco, 04960, Mexico City, Mexico;
3. Facultad de Ciencias, Universidad Nacional Autónoma de México, 04510, Mexico City, Mexico
4. Department of Biochemical and Molecular Biology, Federal University of Santa Maria, Santa Maria, Brazil.
5. School of Health Sciences, Purdue University, West Lafayette, IN, 47907, United States

**
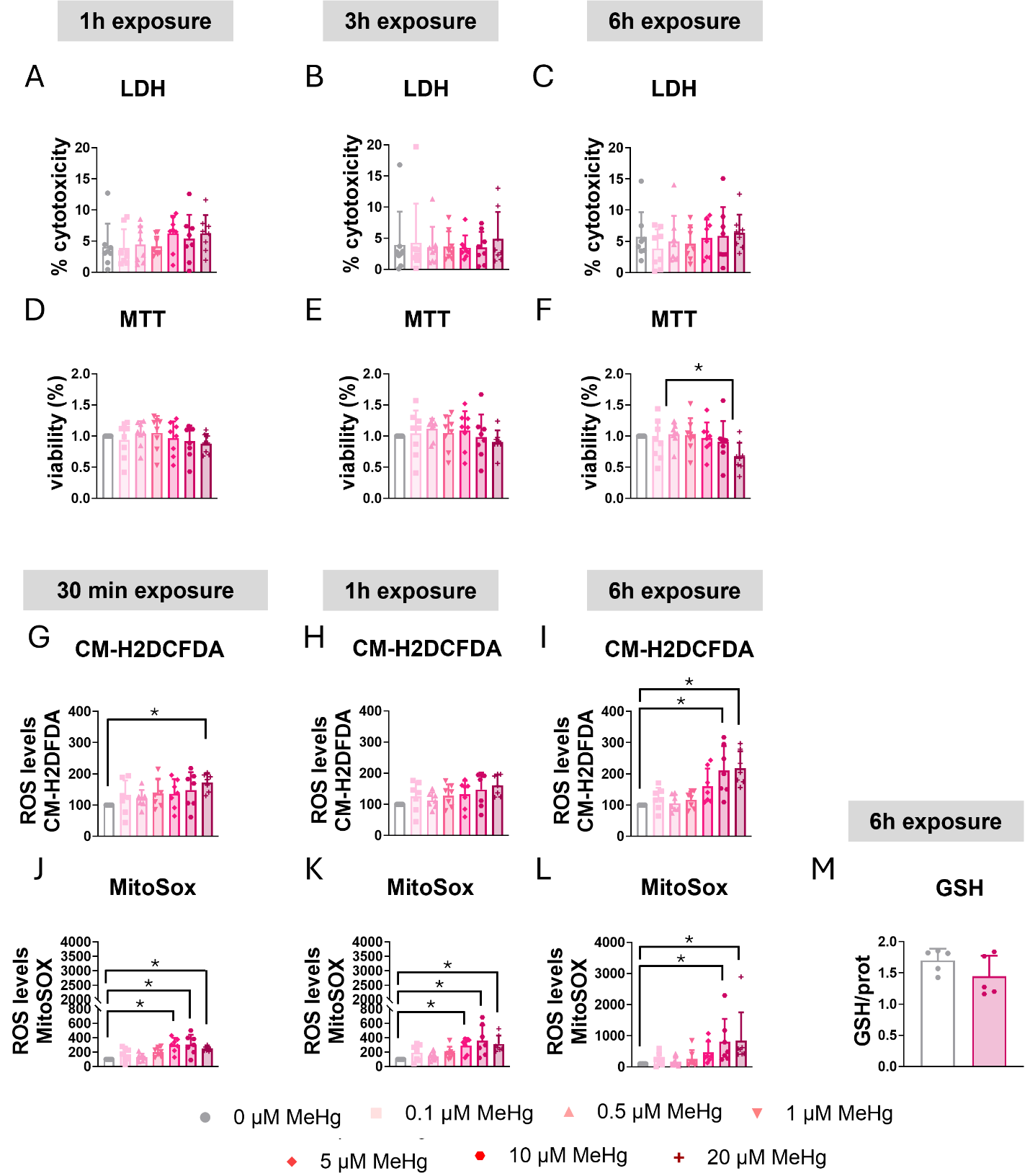
**

**Supplementary Figure 1**. MeHg induced oxidative stress in C8-D1A astrocytic cells. Astrocytic C8-D1A cells were treated with MeHg (0, 0.1, 0.5, 1, 5, 10, or 20 µM). Cytotoxicity (**A**-**C**) and cell viability (**D**-**E**) were measured at 1 hour (**A**-**D**), 3 hours (**B**-**E**), and 6 hours (**C**-**F**) of exposure using LDH and MTT assays, respectively. Total ROS production was measured using the CM-H2DCFDA probe at 30 minutes (**G**), 1 hour (**H**), and 6 hours (**I**). Mitochondrial ROS production was measured using the MitoSOX probe at 30 minutes (**J**), 1 hour (**K**), and 6 hours (**L**) of exposure. (**M**) GSH levels were measured at 6 hours of 10 µM MeHg exposure. Data are presented as mean ± SD. Statistical significance was determined using Mann-Whitney U test when we compared two independent groups (GSH data). When we had more than three independent groups, the statistical significance was determined using one-way ANOVA followed by Bonferroni’s post-hoc analysis, or with the Kruskal-Wallis test followed by Dunn’s post hoc test, adjusted by Bonferroni correction when normality was not achieved. p < 0.05 was considered statistically significant. * denotes a significant difference.


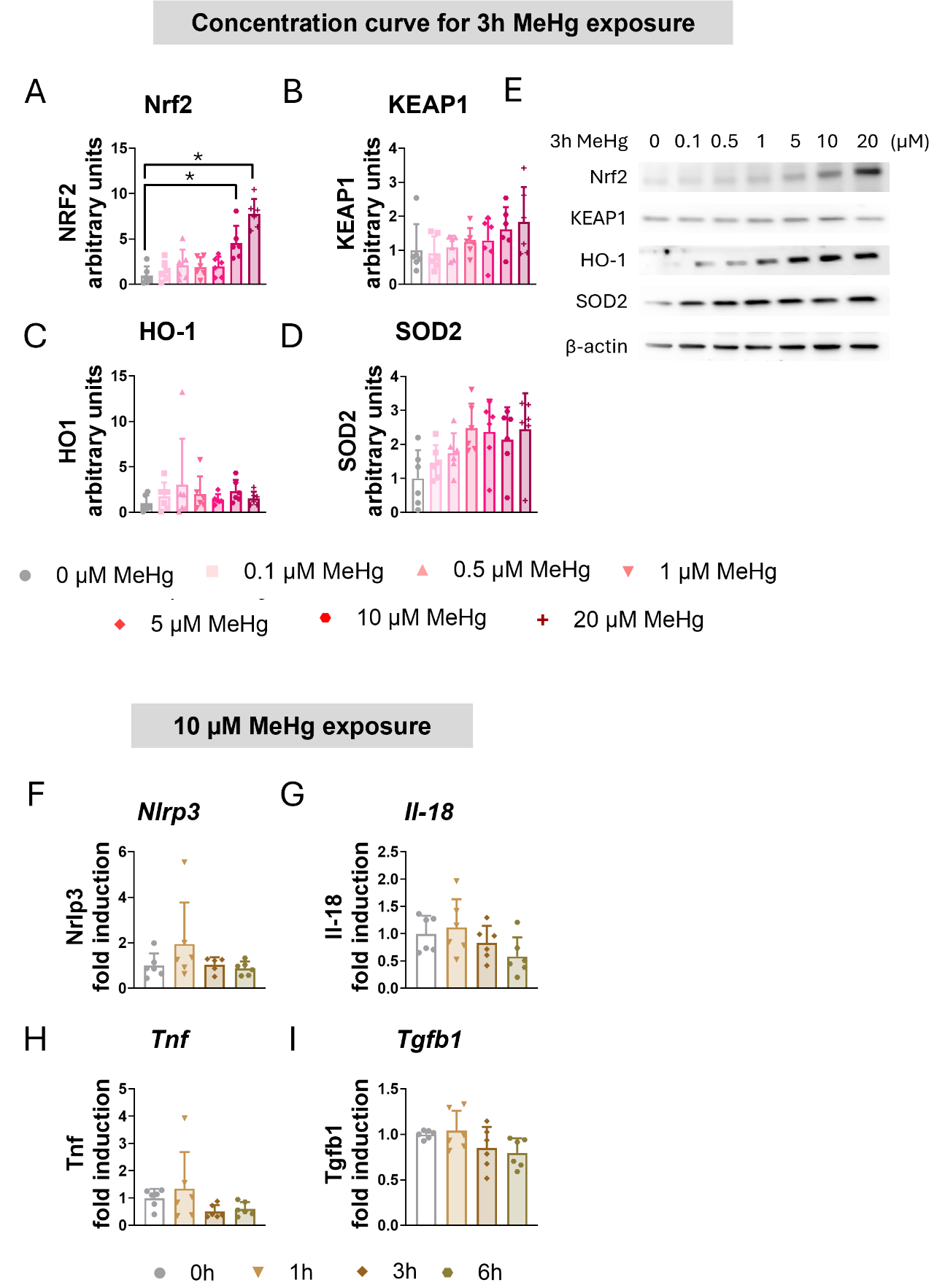
**Supplementary Figure 2**. MeHg induced the expression of antioxidant enzymes in C8-D1A astrocytic cells. Astrocytic C8-D1A cells were treated with MeHg (0, 0.1, 0.5, 1, 5, 10, or 20 µM). Western blot analysis was used to measure changes in the antioxidant proteins Nrf2 (**A**), KEAP1 (**B**), HO-1 (**C**), and SOD2 (**D**) in C8-D1A cells exposed to 0, 0.1, 0.5, 1, 5 ,10, or 20 µM MeHg for 3 hours. (**E**) shows representative densitometry images. *Nlrp3* (**F**), *Il-18* (**G**), *Tnf* (**H**), and *Tgfb1* (**I**) gene expression were measured using qPCR in cells exposed to 10 µM MeHg for 1, 3, or 6 hours. Data are presented as mean ± SD. Statistical significance was determined using Mann-Whitney U test when we compared two independent groups (GSH data). When we had more than three independent groups, the statistical significance was determined using one-way ANOVA followed by Bonferroni’s post-hoc analysis, or with the Kruskal-Wallis test followed by Dunn’s post hoc test, adjusted by Bonferroni correction when normality was not achieved. p < 0.05 was considered statistically significant. * denotes a significant difference.


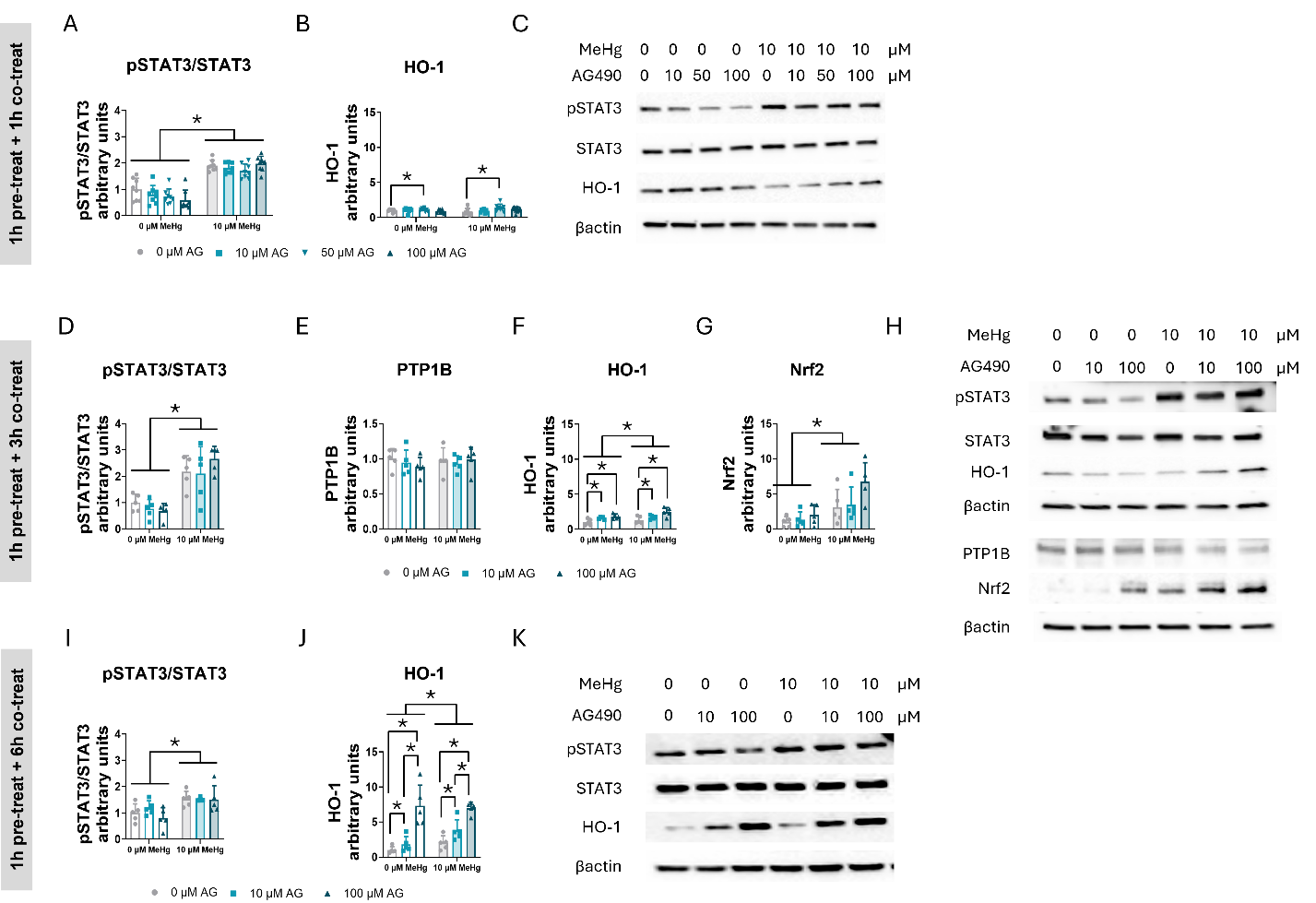


**Supplementary Figure 3**. AG490 inhibitor induced HO-1 expression in in C8-D1A astrocytic cells. Cells were pretreated with 0, 10, 50, or 100 µM AG490 inhibitor, followed by co-treatment with 0 or 10 µM MeHg and protein levels were measured by western blot. (**A**) phosphorylated STAT3 to total STAT3 ratio (pSTAT3/STAT3) and (**B**) HO-1 protein levels 1 hour after adding MeHg. (**C**) Representative densitometry images (1 hour MeHg exposure). (**D**) pSTAT3/STAT3, (**E**) PTP1B, (**F**) HO-1, and (**G**) Nrf2 protein levels 3 hours after adding MeHg. (**H**) Representative densitometry images (3 hours MeHg exposure). (**I**) pSTAT3/STAT3 and (**J**) HO-1 protein levels 6 hours after adding MeHg. (**K**) Representative densitometry images (6 hours MeHg exposure). Data are presented as mean ± SD. Statistical significance was determined using two-way ANOVA followed by Bonferroni’s post-hoc analysis. When data did not meet the assumptions of normality, a logarithmic or square root transformation was employed before conducting the two-way ANOVA. p < 0.05 was considered statistically significant. * denotes a significant difference.


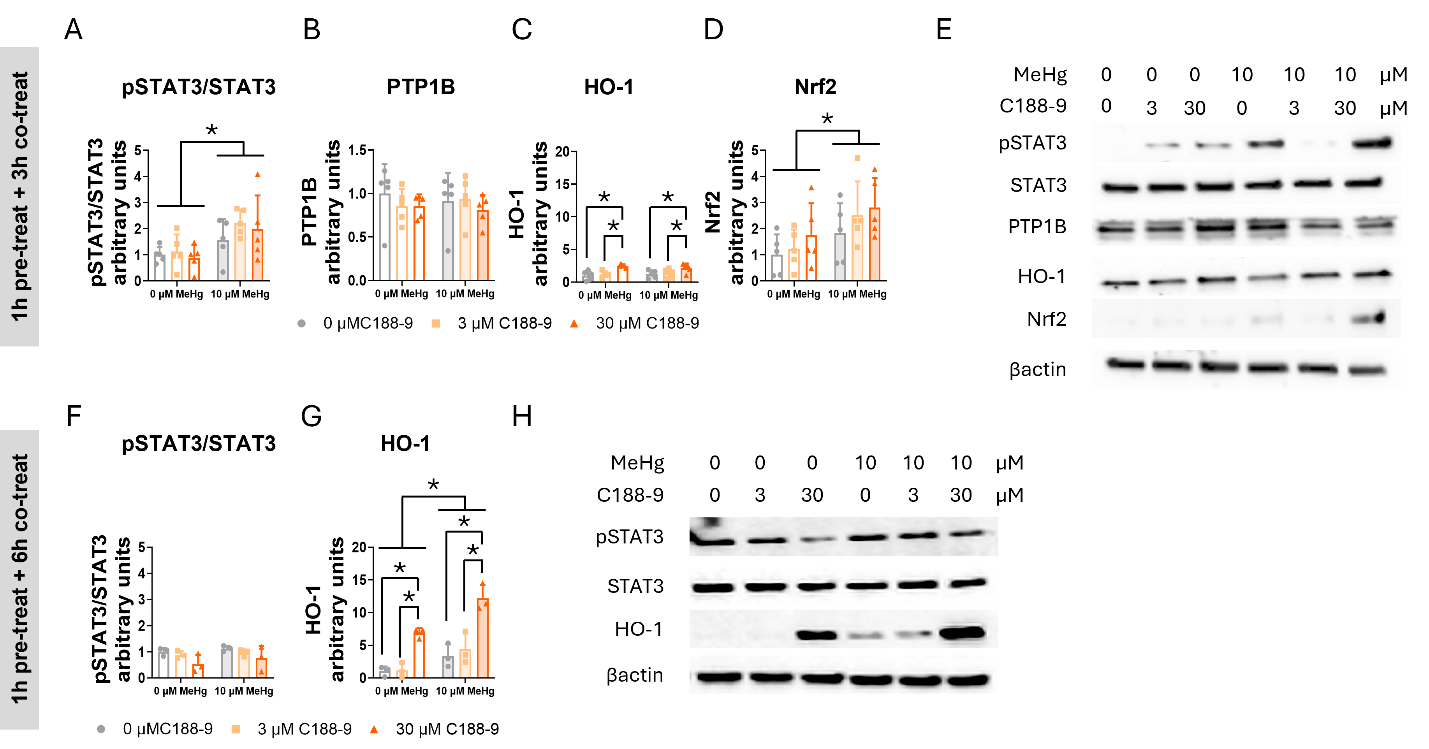


**Supplementary Figure 4**. C188-9 inhibitor induced HO-1 expression in in C8-D1A astrocytic cells. Cells were pretreated with 0, 3, or 30 µM C188-9 inhibitor, followed by co-treatment with 0 or 10 µM MeHg and protein levels were measured by western blot. (**A**) phosphorylated STAT3 to total STAT3 ratio (pSTAT3/STAT3), (**B**) PTP1B, (**C**) HO-1, and (**D**) Nrf2 protein levels 3 hours after adding MeHg. (**E**) Representative densitometry images (3 hours MeHg exposure). (**F**) pSTAT3/STAT3 and (**G**) HO-1 protein levels 6 hours after adding MeHg. (**H**) Representative densitometry images (6 hours MeHg exposure). Data are presented as mean ± SD. Statistical significance was determined using two-way ANOVA followed by Bonferroni’s post-hoc analysis. When data did not meet the assumptions of normality, a logarithmic or square root transformation was employed before conducting the two-way ANOVA. p < 0.05 was considered statistically significant. * denotes a significant difference.

**
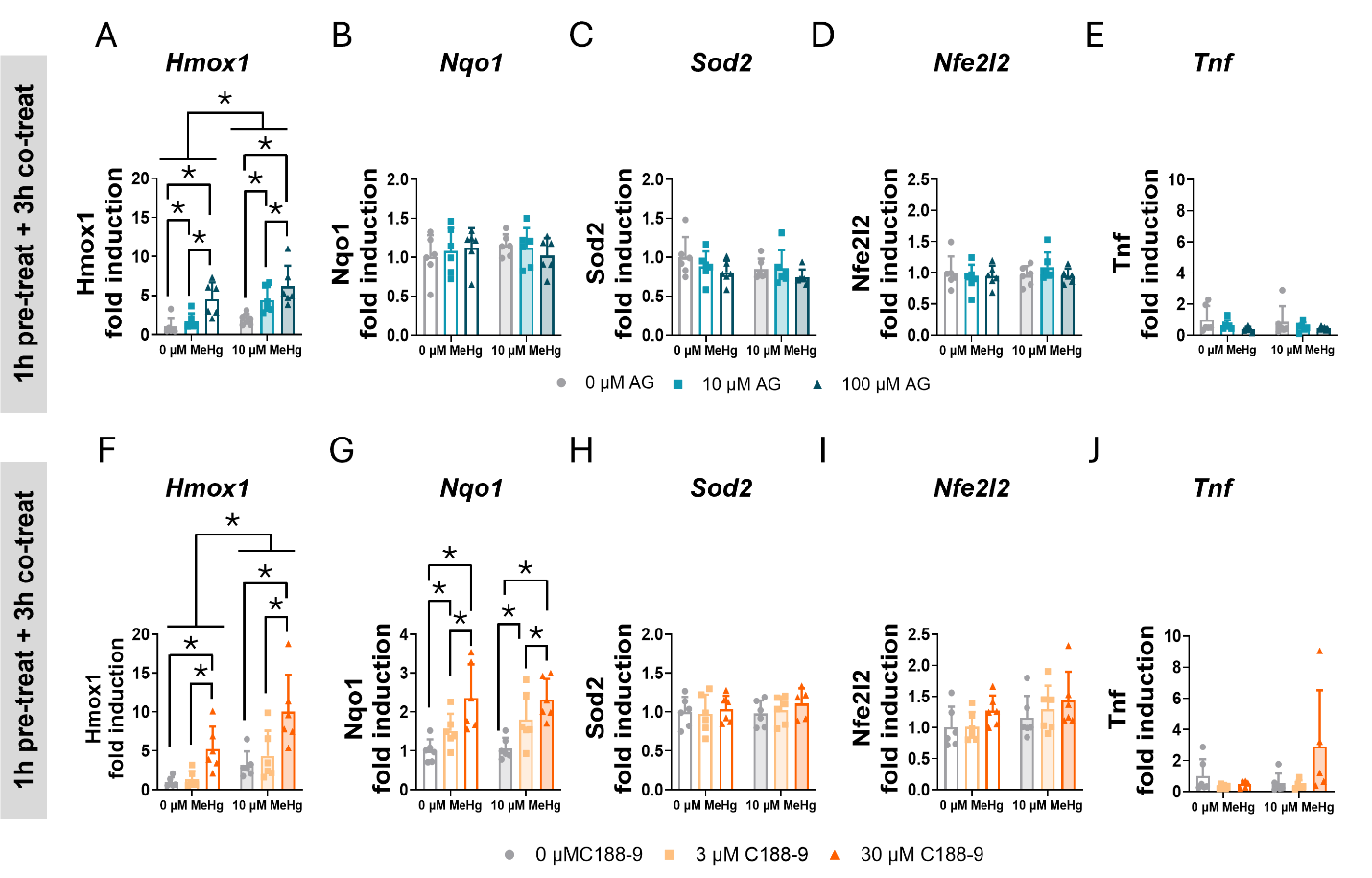
**

**Supplementary Figure 5**. AG490 inhibitor induced HO-1 expression in in C8-D1A astrocytic cells. Cells were pretreated with 0, 10, or 100 µM AG490 inhibitor (**A**-**E**) or with 0, 3, or 30 µM C188-9 inhibitor (**F**-**J**), followed by co-treatment with 0 or 10 µM MeHg for 3 hours and (**A**, **F**) *Hmox1*, (**B**, **G**) *Nqo1*, (**C**, **H**) *Sod2*, (**D**, **I**) *Nfe2l2*, and (**E**, **J**) *Tnf* gene expression was measured using qPCR. Data are presented as mean ± SD. Statistical significance was determined using two-way ANOVA followed by Bonferroni’s post-hoc analysis. When data did not meet the assumptions of normality, a logarithmic or square root transformation was employed before conducting the two-way ANOVA. p < 0.05 was considered statistically significant. * denotes a significant difference.

**
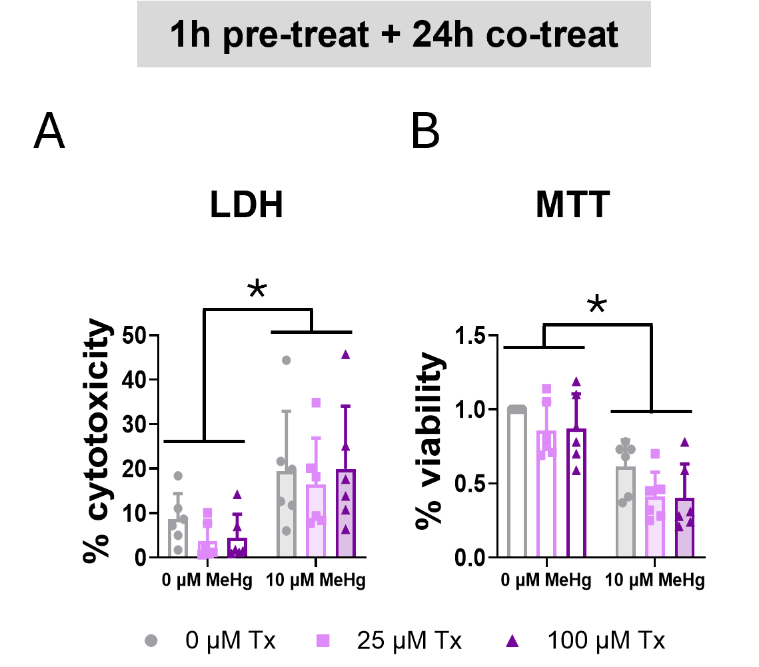
**

**Supplementary Figure 6.** Trolox failed to counteract the MeHg-induced mortality. Cells were pretreated with 0, 25, or 100 µM Trolox, followed by co-treatment with 0 or 10 µM MeHg. (**A**) Cytotoxicity was measured using LDH assay after 24 hours of MeHg addition. (**B**) Cell viability was assessed by MTT assay after 24 hours of MeHg addition.. Data are presented as mean ± SD. Statistical significance was determined using two-way ANOVA followed by Bonferroni’s post-hoc analysis. When data did not meet the assumptions of normality, a logarithmic or square root transformation was employed before conducting the two-way ANOVA. p < 0.05 was considered statistically significant. * denotes a significant difference.

**
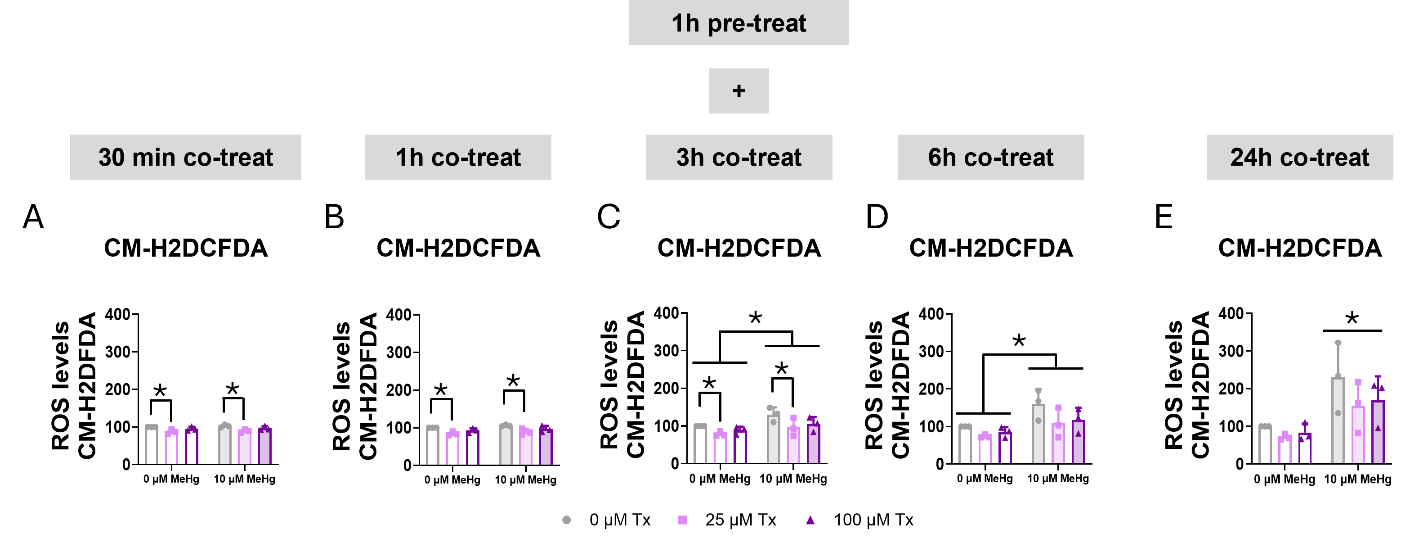
**

**Supplementary Figure 7.** Trolox failed to counteract the MeHg-induced oxidative stress. Cells were pretreated with 0, 25, or 100 µM Trolox, followed by co-treatment with 0 or 10 µM MeHg. ROS levels were measured using CM-H2DCFDA probe at 30 minutes (**A**), 1 hour (**B**), 3 hours (**C**), 6 hours (**D**), and 24 hours (**E**) after MeHg addition. Data are presented as mean ± SD. Statistical significance was determined using two-way ANOVA followed by Bonferroni’s post-hoc analysis. When data did not meet the assumptions of normality, a logarithmic or square root transformation was employed before conducting the two-way ANOVA. p < 0.05 was considered statistically significant. * denotes a significant difference.

**
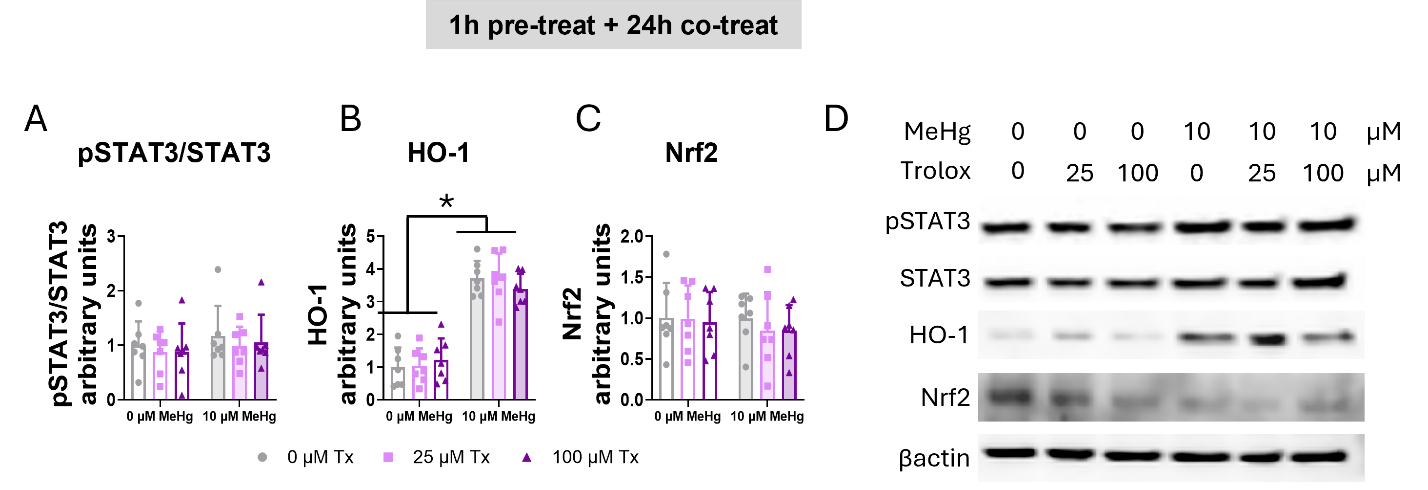
**

**Supplementary Figure 8.** Trolox failed to counteract MeHg-induced expression of antioxidant enzymes in C8-D1A astrocytic cells. Cells were pretreated with 0, 25, or 100 µM Trolox, followed by co-treatment with 0 or 10 µM MeHg. (**A**) phosphorylated STAT3 to total STAT3 ratio (pSTAT3/STAT3), (**B**) HO-1, and (**C**) Nrf2 protein levels were measured by western blot 24 hours after MeHg addition. (**D**) shows representative densitometry images. Data are presented as mean ± SD. Statistical significance was determined using two-way ANOVA followed by Bonferroni’s post-hoc analysis. When data did not meet the assumptions of normality, a logarithmic or square root transformation was employed before conducting the two-way ANOVA. p < 0.05 was considered statistically significant. * denotes a significant difference.
